# Intravital Multiphoton Microscopy of the Ocular Surface: Alterations in Conventional Dendritic Cell Morphology and Kinetics in Dry Eye Disease

**DOI:** 10.3389/fimmu.2020.00742

**Published:** 2020-05-07

**Authors:** Arsia Jamali, Yashar Seyed-Razavi, Cecilia Chao, Gustavo Ortiz, Brendan Kenyon, Tomas Blanco, Deshea L. Harris, Pedram Hamrah

**Affiliations:** ^1^Center for Translational Ocular Immunology, Tufts Medical Center, Tufts University School of Medicine, Boston, MA, United States; ^2^Department of Ophthalmology, Tufts Medical Center, Tufts University School of Medicine, Boston, MA, United States; ^3^Program in Neuroscience, School of Graduate Biomedical Sciences, Tufts University, Boston, MA, United States; ^4^Program in Immunology, School of Graduate Biomedical Sciences, Tufts University, Boston, MA, United States

**Keywords:** intravital imaging, multiphoton microscopy, conventional dendritic cells, ocular surface, dry eye disease, cornea, limbus, sensory nerves

## Abstract

Dry eye disease (DED) is a multifactorial disease of the ocular surface, characterized by loss of tear film homeostasis and ocular symptoms, in which neurosensory abnormalities have recently been shown to play an etiological role. Although the role of inflammation has been widely studied in DED, the kinetics of immune cells of the ocular surface in this complex disease are hereto unclear. Herein, we utilized intravital multiphoton imaging on transgenic mice to investigate the 3D morphology and kinetics of conventional dendritic cells (cDCs) and the role of ocular surface sensory nerves in regulating them in both the naïve state and experimental DED. Mice with DED had significantly lower tear secretion (*p* < 0.01), greater corneal fluorescein staining (*p* < 0.001), and higher cDC density in the ocular surface (*p* < 0.05), compared to naïve mice. cDCs in DED mice showed morphological alterations in the limbus, exhibiting smaller surface area (*p* < 0.001) and volume (*p* < 0.001) compared to naïve mice. Furthermore, corneal cDCs showed greater sphericity in DED mice compared to naïve mice (*p* < 0.01). In addition, limbal cDCs displayed significantly increased migratory kinetics in DED, including mean track speed, 3D instantaneous velocity, track length, and displacement, compared to naïve mice (all *p* < 0.05). In mice with DED, cDCs showed a higher meandering index in the limbus compared to central cornea (*p* < 0.05). In DED, cDCs were less frequently found in contact with nerves in the limbus, peripheral, and central cornea (*p* < 0.05). cDCs in contact with nerves demonstrated a larger surface area (*p* < 0.001) and volume (*p* < 0.001), however, they exhibited less sphericity (*p* < 0.05) as compared to cDCs not in contact with nerves in naïve mice. Importantly, cDCs in contact with nerves during DED had a decreased track length, displacement, mean track speed, and 3D instantaneous velocity compared to those not in contact with nerves (all *p* < 0.05). Taken together, we present *in vivo* evidence of altered cDC kinetics and 3D morphology in DED. Furthermore, apparent neuronal contact significantly alters cDC kinetics and morphological characteristics, suggesting that ocular surface nerves may play a direct role in mediating immune responses in DED.

## Introduction

Dry eye disease (DED) is one of the most common causes of clinical ophthalmic visits, since it is accompanied by ocular discomfort and results in diminished quality of life (1). It is estimated that more than 16 million adults in the United States suffer from DED, with an estimated prevalence of 3.0% in men and 7.8% in women, imposing a significant public health and economic burden (2, 3). DED is “a multifactorial disease of the ocular surface characterized by a loss of homeostasis of the tear film, and accompanied by ocular symptoms, in which tear film instability and hyperosmolarity, ocular surface inflammation and damage, and neurosensory abnormalities play etiological roles” (1). It can arise due to desiccating stress, systemic conditions, medications, and neurosensory abnormalities (1).

The ocular surface is composed of the avascular cornea, neighboring conjunctiva, eyelids, tear film, and secretory glands, including lacrimal and meibomian glands. The ocular surface functions as a physical and immunological barrier to the external environment, such as foreign particles and opportunistic pathogens while allowing light to penetrate into the eye by maintaining the health of the ocular surface and tear film stability. The cornea is densely innervated by sensory nerves supplied by the ophthalmic division of the trigeminal nerve, sympathetic fibers derived from the superior cervical ganglion, and parasympathetic fibers that originate from the ciliary ganglion (4, 5). Sensory nerves enter the peripheral cornea in a radial pattern and travel parallel to the corneal surface toward the corneal center. The nerve fibers in the stroma comprise the stromal nerve plexus and the branches beneath the basal epithelial cell layer form a dense nerve plexus, termed the subbasal nerve plexus (4, 6). In addition to its dense innervation, the cornea is an immune privileged tissue and also hosts antigen-presenting cells (APCs), including epithelial and stromal conventional dendritic cells (cDCs) and stromal macrophages during steady state (7–12).

Involved in both initiation and progression of the DED, inflammation is considered a key process in the pathogenesis of DED (13–16). The inflammatory microenvironment in the ocular surface during DED facilitates maturation of ocular surface APCs and their subsequent egress to the draining lymph nodes, where they prime naïve CD4^+^ T cells toward effector T helper (Th)1 and Th17 cells (17–19). Through the efferent arm of the immune response, effector T cells migrate to the ocular surface, where they exacerbate inflammation via further tissue damage. In addition, DED-associated inflammation further exacerbates the destructive milieu in the ocular surface, leading to decreased goblet cell density, altered tear composition, and increased surface irregularity, generating focal points for accelerated tear thinning, and consequently promoting squamous metaplasia in an interferon gamma (IFN-γ)-mediated manner (20, 21). Furthermore, inflammation directly stimulates corneal nociceptor nerve terminals (22, 23). Persistent ocular surface inflammation then results in nerve fiber damage, which in turn perpetuates the disease (24). In this regard, it has been shown that desiccating stress leads to diminished corneal subbasal and intraepithelial nerve density, altered nerve morphology, decreased cornea sensitivity, and pain (25–30). Damage and malfunction of the corneal sensory nerves can lead to decreased blink reflex and tear production, which further augments the disease (31–33).

Although the role of inflammation in the pathogenesis of DED is well studied (14–17, 34), our knowledge on the kinetics of corneal immune cells and the potential role of nerves in affecting immune cell migration during the course of DED is limited. In this study, we investigate the effects of desiccating stress-induced DED on alterations in the three-dimensional (3D) morphology and kinetics of cDCs, as well as their relation to nerves. Utilizing intravital multiphoton microscopy (IV-MPM) of the ocular surface of transgenic mice with fluorescent-tagged cDCs and nerves, we demonstrate that cDCs are more spherical and more motile during DED. In addition, we demonstrate how contact with nerves impacts these morphological and kinetic alterations of cDCs during DED.

## Materials and Methods

### Mice

Wild-type female C57BL/6 mice were purchased from Charles River (Charles River Laboratories, Wilmington, MA, United States). Thy1^YFP^ mice [B6.Cg-Tg (Thy1-YFP)16Jrs/J] were obtained from the Jackson Laboratory (Bar Harbor, ME, United States) as heterozygous and bred to homozygous with repeated matings between male and female mice with high copies of the transgenes for yellow fluorescent protein (YFP). This was required in order to obtain mice with higher fluorescence for IV-MPM. CD11c^EYFP^ mice were a generous gift from Dr. Michel C. Nussenzweig from Rockefeller University (35). CD11c^YFP^ × Thy1^YFP^ mice were generated by crossing homozygous Thy1^YFP^ with homozygous CD11c^EYFP^ repeatedly until both the nerves and cDCs were colocalized with YFP in the cornea. Primer sets used for quantitative PCR for genotyping included the following: Thy1-YFP forward 5-GCCCTGGCCCACCCTCGTGACCACCTTCG-3, Thy1-YFP reverse 5- CCTGATGCCGTTCTTCTGCTTGTCGGGCA-3, CD11c-EYFP forward 5-TGCTGGTTGTTGTGCTGTCTCATC- 3, and CD11c-EYFP reverse 5-GGGGGTGTTCTGCTGGTAG TGGTC-3.

The Thy1-YFP mice express YFP under the control of regulatory elements of the Thy1 gene and thus label neuronal populations, primarily sensory and motor neurons. The CD11c-YFP mice carry the EYFP transgene under the control of the CD11c promoter (35). Thus, our CD11c^YFP^ × Thy1^YFP^ mice allow visualization of both CD11c^+^ cDCs and Thy1^+^ neurons in the same animals. Because females are more prone to DED and female mice develop more severe clinical disease than age-matched males (36), in this study, only 6- to 8-week-old female mice were used in all experiments. Mice were housed at Tufts Department of Lab Animal Medicine and were treated in accordance with the Association of Research and Vision in Ophthalmology (ARVO) statement for the Use of Animals in Ophthalmology and Vision Research. All experiments were performed after the review and approval from the Institutional Animal Care and Use Committee (IACUC number B2018-47) at Tufts University and Tufts Medical Center, Boston, MA, United States.

### Murine Model of Dry Eye Disease

At the age of 6–8 weeks, CD11c^YFP^ × Thy1^YFP^ mice were either housed in a low-humidity, temperature-regulated custom-made controlled environment chamber (Percival Scientific Inc, Perry, IA, United States) for 4 weeks to develop desiccating stress-induced evaporative DED, or were kept under standard housing with a humidity of 50–60% and a temperature of 21–23°C to serve as naïve controls (37). Using the controlled environment chamber, we exposed the animals to a humidity of 15%, and air circulation of 15 L/min, at 21–23°C temperature using the INTELLIS Ultra Control System and Desiccant dryer 50 cfm leading to desiccating stress. The chamber is sealed, avoiding the direct exchange of air between the outside and the inside, and is connected to a desiccant, which introduces air with low humidity inside the chamber. Inside the chamber, three sensors are located in order to monitor the humidity, airflow, and temperature. Sensors are connected to a router in order to automatically monitor the parameters. In order to maximize exposure, mice were housed in custom-designed fenestrated cages (Ancare Corp. Bellmore, NY, United States). The cages were built with vents at each side to maximize the airflow through them in order to achieve greater desiccating stress on the ocular surface.

### Clinical Evaluation

Corneal fluorescein staining (CFS) was performed and scored according to the National Eye Institute (NEI) scale (38). In brief, CFS was graded in five corneal regions, each ranging from 0 to 3, and the sum of the scores of all regions was measured (range, 0–15). Tear secretion was assessed by measuring the wetted length of a phenol red thread (Zone-Quick; Hilco Vision Headquarters, Plainville, MA, United States), which was placed in the lateral canthus of anesthetized mice for 30 sec (39, 40).

### Ocular Surface Intravital Multiphoton Microscopy

Animals were anesthetized, and body and ocular surface temperatures were tightly regulated as previously described (41, 42). For central corneal IV-MPM, following intraperitoneal injection of ketamine (100 mg/kg), xylazine (20 mg/kg), and acepromazine (3 mg/kg) mixture, mice were placed on a custom and tiltable stereotactic stage to focus on the cornea and limbus. Proper depth of anesthesia was assessed every 30–60 min with supplemental doses of ketamine alone (100 mg/kg). Topical proparacaine hydrochloride (0.5%) and Genteal ophthalmic lubricant gel (Alcon, Fort Worth, TX, United States) were applied to the examined eye and a 5-0 non-absorbable silk suture (Surgical Specialities Corporation, Reading, PA, United States) was placed around the eye to prevent eye closure, blinking, and nystagmus while allowing the normal flow of blood to the eye. To maintain ocular surface temperature and moisture, a sealed chamber was created around the exposed portion of the eye using high vacuum grease (Dow Corning, Midland, MI, United States) and plastics placed on the grease as previously described (41). Genteal gel was added to the chamber, and hot water circulated through the tubing and chamber assembly to maintain a temperature range within 2°C of the physiological ocular surface temperature (34°C; range, 33-35°C). The IV-MPM setup and surgical mouse preparation for imaging the limbus and peripheral cornea are described elsewhere (42).

An Ultima Multiphoton Microscope System (Bruker, Fitchburg, WI, United States) equipped with two MaiTai Ti/Sapphire DeepSee lasers (Newport Spectra-Physics, Irvine, CA, United States), allowing for simultaneous coaxial 850 and 900 nm illumination to achieve two-photon excitation and second harmonic generation, was utilized for all experiments. Using a 20 × −1.0 NA (Olympus XLUMPLFLN, Tokyo, Japan) water immersion objective, scans of the ocular surface were taken every 30–60 sec over a period of up to an hour with 512 × 512 resolution and twofold line averaging, as previously described (41).

### Image Analysis and Processing

3D measurements of cell morphology and cell motility were calculated using Imaris (Bitplane, Zurich, Switzerland) as previously described (41). In short, 3D cell analysis was performed to elucidate 3D cell surface area (μm^2^), volume (μm^3^), and sphericity (range, 0–1) by creating a surface object using the surface tool. Careful consideration and manual confirmation of each cell surface was performed to ensure that created surfaces were representative of the morphology of individual cells. Image stacks were converted into four-dimensional (4D) movies, semi-automated tracking of cell motility was performed, and cell bodies were tracked over time and were manually confirmed at each frame to determine total track length (total track distance of a cell, μm) and displacement length (distance from the initial position of a cell in the track to the last, as a straight line, μm), 3D instantaneous velocity (velocity of a cell between two consecutive frames, μm/min), mean track speed (average speed of a cell over length of imaging, μm/min), and meandering index, which provides a measure of the deviation from a straight line of a migratory cell (a value of 1 indicating that the track is a straight line) (43). Static cells with a displacement of <10 μm were excluded from meandering index analysis (41). In order to elucidate the effect of cDC contact with nerves on cDC morphological and kinetic parameters, each cell in all videos from all areas of limbus, peripheral, and central cornea was assessed by two independent investigators using 3D and 4D reconstructed videos to evaluate the presence of contact or lack of contact with nerves (interobserver agreement kappa = 0.83). All cells in the assessed videos were categorized as either with or without contact with nerves, unless the investigators did not agree on the presence or lack of contact with nerves. In such cases, the examined cells were excluded from further analysis on the effect of nerve contact on cDC morphology and kinetics.

### Confocal Microscopy

In order to assess if we can observe cDCs in contact with nerves in the limbus and cornea of wild-type C57BL/6 and CD11c^YFP^ × Thy1^YFP^ mice, the corneas and limbus of wild-type C57BL/6 and CD11c^YFP^ × Thy1^YFP^ mice were excised; samples from wild-type mice were fixed in chilled acetone for 15 min, washed, blocked for 30 min at room temperature (RT) with 3% bovine serum albumin (BSA) containing 1% anti-CD16/CD32 Fc receptor mAb (Bio X Cell, West Lebanon, NH, United States), and were then incubated with a combination of primary fluorophore-conjugated antibodies against Thy1, CD45, F4/80 (all Biolegend, San Diego, CA, United States), and CD11c (eBioscience, San Diego, CA, United States) for 90 min at RT. After washing for three times, samples were mounted with mounting media containing 4′,6-diamidino-2-phenylindole (DAPI; Vector Laboratories, Inc., Burlingame, CA, United States) and were visualized by a Leica SP8 confocal microscope (Leica Microsystems Inc., Buffalo Grove, IL, United States). Samples from CD11c^YFP^ × Thy1^YFP^ mice were freshly mounted and underwent confocal microscopy. After acquisition, the epithelium was digitally removed, and 3D videos were reconstructed using Imaris.

### Flow Cytometry

The bone marrow of CD11c^YFP^ × Thy1^YFP^ double-transgenic mice were harvested and strained using a 70 μm nylon mesh to yield single-cell suspension. Red blood cells were removed by incubating the single cells in ammonium-chloride-potassium lysing buffer for 1 min at RT. Samples were then washed, blocked and stained in fluorescence-activated cell sorting buffer containing 1% anti-CD16/CD32 Fc receptor mAb (Bio X Cell) and LIVE/DEAD Fixable Blue Dead Cell Stain (Thermo Fisher Scientific, Waltham, MA, United States) at RT for 20 min. Next, single cell suspensions were labeled with fluorophore-conjugated antibodies against CD45, CX3CR1, CD11c, CD11b, CD68, and PDCA-1, or appropriate conjugated isotype controls, for 60 min at RT (all Biolegend or eBioscience). Following a wash, samples underwent flow cytometric analysis using a BD LSR II Flow Cytometer (BD Biosciences). The acquired data were analyzed by FlowJo v10 (FlowJo, LLC, Ashland, OR, United States). Debris, dead cells, and doublets were excluded using forward and side scatters and a live/dead cell marker (Supplementary Figure S1).

### Statistical Analysis

Results are presented as mean ± standard error of the mean (SEM). To compare cDC morphological and kinetic characteristics between groups, at least three IV-MPM videos, each taken from an individual mouse, were pooled. To determine the differences in cDC density, morphological and kinetic parameters between experimental DED and naïve groups, as well as between cDCs in contact and not in contact with nerves, *t* test was employed. To assess regional differences in the density, morphology, and kinetic parameters of cDCs (in three regions of limbus, peripheral, and central cornea), ANOVA with Tukey *post hoc* test was used (Prism GraphPad Software, La Jolla, CA, United States). Differences between groups were considered significant if *p* < 0.05.

## Results

### Clinical Assessment of Dry Eye Disease Severity

**FIGURE 1:**
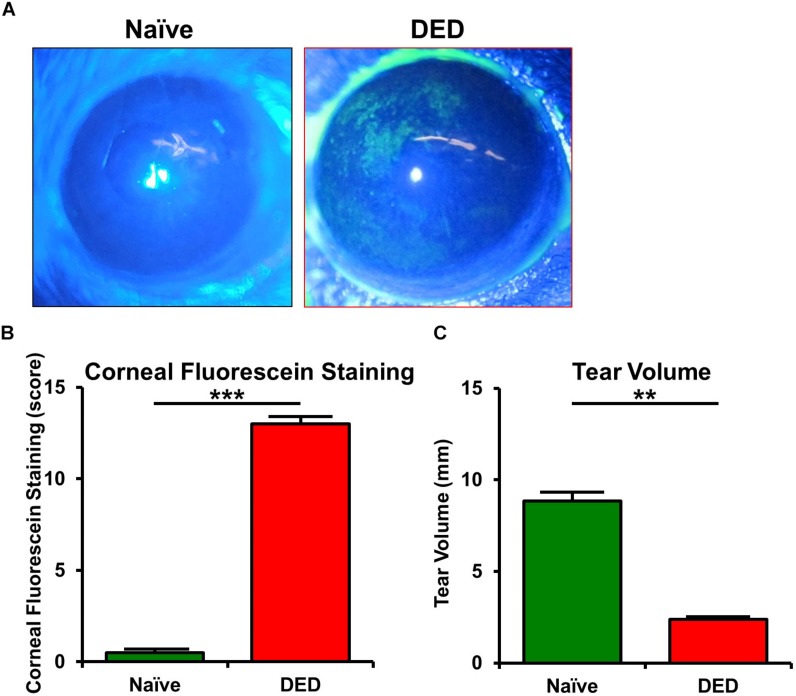
Ocular surface findings in desiccating stress-induced dry eye disease. **(A)** Representative corneal fluorescein staining images. **(B)** Quantification of fluorescein staining scores revealed a significant increase in corneal staining in mice exposed to desiccating stress compared to naïve controls (*n* = 5–6/group). **(C)** Tear volume using phenol red thread test showed a significant decrease in tear volume in mice exposed to desiccating stress compared to naïve controls (*n* = 6–12/group). Results are presented as mean ± SEM, *t* test, **p < 0.01, ***p < 0.001.

Clinical assessment revealed that mice exposed to desiccating stress developed clinical signs of DED, with a significantly higher CFS (13.8 ± 0.3) compared to naïve controls (0.5 ± 0.2, *p* < 0.001; *n* = 5–6/group; Figures 1A,B). Furthermore, the mice exposed to desiccating stress showed lower tear volume (3.4 ± 0.92 mm) in comparison to naïve mice (8.8 ± 0.5 mm, *p* = 0.005; *n* = 6–12/group; Figure 1C).

### Alterations in Dendritic Cell Density and 3D Morphology in Dry Eye Disease

We initially aimed to characterize the identity of YFP^+^ cells in CD11c^YFP^ × Thy1^YFP^ mice. Thus, we performed flow cytometry on the bone marrow of these transgenic mice. As depicted in Supplementary Figure S1, we initially gated out debris and dead cells (Supplementary Figure S1A), doublets (Supplementary Figure S1B), and gated on YFP^+^ cells (Supplementary Figure S1C). Next, using fluorescent minus one controls, we observed that YFP^+^ cells in the bone marrow of CD11c^YFP^ × Thy1^YFP^ mice were in fact cDCs, as they expressed the pan-leukocyte marker, CD45, myeloid markers, CD11b and CX3CR1, dendritic cell marker, CD11c; but were majorly negative for plasmacytoid dendritic cell marker, PDCA-1, as well as macrophage marker, CD68 (Supplementary Figure S1D).

**FIGURE 2:**
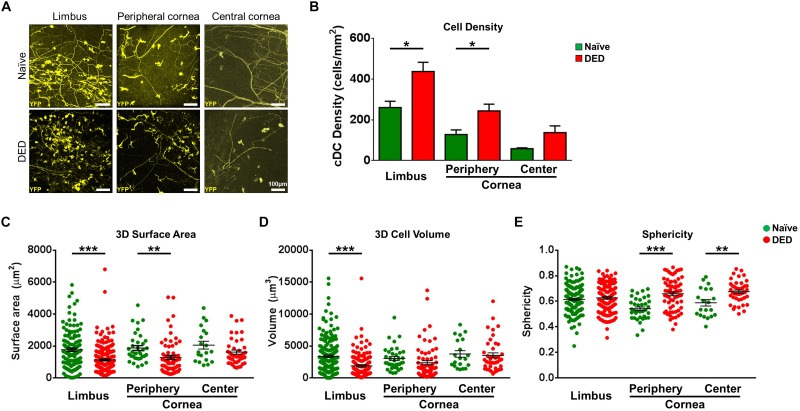
Morphological features of conventional dendritic cells. **(A)** Representative intravital multiphoton microscopy (IV-MPM) images of ocular surface conventional dendritic cells (cDCs) in naïve mice and after exposure to desiccating stress-induced dry eye disease (DED). **(B)** Quantification of the density of YPF^+^ cDCs highlighting an increase in cDCs in the limbus and cornea in DED (*n* = 3–5/group). **(C–E)** 3D Morphological analyses of YPF^+^ cDCs in naïve and after exposure to desiccating stress, pooled from at least three mice/group, including **(C)** 3D cell surface area, **(D)** 3D cell volume, and **(E)** sphericity. Scale bars: 100 μm. Results are presented as mean ± SEM, *t* test, *p < 0.05, **p < 0.01, and ***p < 0.001.

Having established the identity of YFP^+^ cells in CD11c^YFP^ × Thy1^YFP^ mice, we next examined if desiccating stress-induced DED affects the density of YFP^+^ cDCs in the limbus, peripheral, and central cornea, using IV-MPM (Figure 2A). A higher density of YFP^+^ cDCs was found in both the limbus and peripheral cornea in the DED group (limbus, 437.5 ± 45.1 cells/mm^2^; peripheral cornea, 243.8 ± 32.9 cells/mm^2^) compared to naïve controls (limbus, 260.4 ± 30.9 cells/mm^2^, *p* = 0.013; peripheral cornea, 127.5 ± 23.2 cells/mm^2^, *p* = 0.021; Figure 2B). However, despite a 2.4-fold increase, the density of cDCs in the central cornea did not reach statistical significance (137.5 ± 33.1 cells/mm^2^ in DED vs. 58.3 ± 4.2 cells/mm^2^ in naïve mice, *p* = 0.076; Figure 2B). Furthermore, as expected, in both naïve mice and mice with DED, we observed higher densities of cDCs in the limbus compared with that in the peripheral (*p* = 0.027 and *p* = 0.009, respectively) and central corneas (*p* = 0.003 and *p* < 0.001, respectively).

We next compared the 3D morphological parameters of cDCs in DED to naïve mice. As shown in Figure 2C, cDCs in the limbus and peripheral cornea of DED mice were smaller, with lower 3D cell surface area compared to that of naïve mice (limbus, 1,127.2 ± 55.0 vs. 1,761.9 ± 104.1 μm^2^, *p* < 0.001; peripheral cornea, 1,279.0 ± 129.8 vs. 1,875.5 ± 148.5 μm^2^, *p* = 0.005), but there was no difference in the central cornea between the groups (DED, 1,616.5 ± 128.4 vs. naïve mice, 2,049.8 ± 242.8 μm^2^, *p* = 0.088; Figure 2C). cDC volume in the limbus was also lower in DED compared to that in the naïve group (1,880.8 ± 184.3 vs. 3,371.5 ± 186.3 μm^3^, *p* < 0.001; Figure 2D) but not in the peripheral cornea (2,375.4 ± 324.0 vs. 3,038.9 ± 306.5 μm^3^, *p* = 0.184) and central cornea (3,530.7 ± 391.6 vs. 3,750.1 ± 510.4 μm^2^, *p* = 0.744; Figure 2D). Furthermore, assessment of cDC sphericity in DED exhibited higher sphericity in the peripheral (0.66 ± 0.02 vs. 0.54 ± 0.01, *p* < 0.001) and central corneas (0.68 ± 0.01 vs. 0.59 ± 0.03, *p* = 0.002), but not in the limbus (0.63 ± 0.01 vs. 0.62 ± 0.01, *p* = 0.432) compared to naïve controls (Figure 2E). Assessing the potential regional differences in the morphology of cDCs, we noticed that it is only during DED that limbal cDCs exhibited less 3D cell surface area (*p* = 0.035; Supplementary Figure S2A) and volume (*p* = 0.002; Supplementary Figure S2B) compared to central corneal cDCs. However, we did not observe significant regional differences in the sphericity of cDCs during DED (Supplementary Figure S2C).

Taken together, our results highlight that DED significantly alters cDC morphology in the limbus and cornea, with more prominent alterations in the limbus and peripheral cornea.

### Alterations in cDC Kinetics in Dry Eye Disease

**FIGURE 3:**
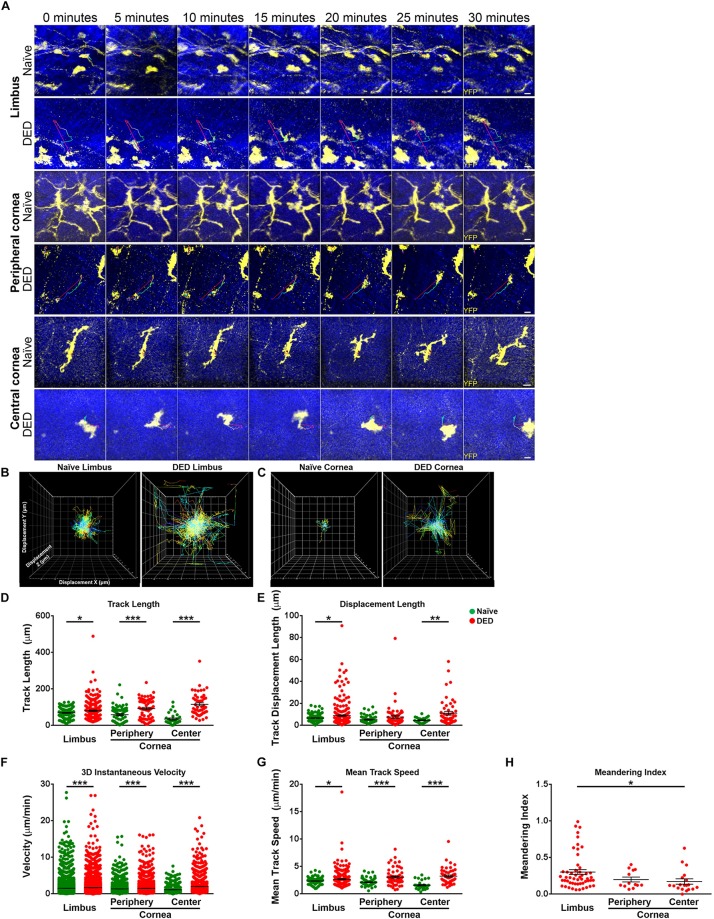
Kinetics of conventional dendritic cells. **(A)** Representative still images of YFP^+^ conventional dendritic cells (cDCs) in the limbus (top panels), peripheral (middle panels), and central corneas (bottom panels). The thin colored lines represent cell tracks based on the time scale. **(B,C)** Representative displacement tracks of YFP^+^ cDCs with centered alignment in the **(B)** limbus and **(C)** cornea in naïve state and after exposure to desiccating stress-induced dry eye disease (DED) reveal altered displacement and directionality of cDCs after exposure to desiccating stress. **(D,E)** Kinetic characteristics including **(D)** track length, **(E)** displacement length, **(F)** 3D instantaneous velocity, **(G)** mean track speed, and **(H)** and meandering indices of YFP^+^ cDC tracks, pooled from at least three mice/group. Scale bars: 10 μm. Results are presented as mean ± SEM, *t* test, *p < 0.05, **p < 0.01, and ***p < 0.001.

Having observed alterations in the morphological properties of cDCs in DED, we next investigated if the *in vivo* ocular surface cDC kinetics are altered in DED. Thus, we performed IV-MPM and analyzed kinetics of cDCs in the limbus/peripheral corneas of naïve mice (Supplementary Video S1) and mice with DED (Supplementary Video S2), as well as in the central cornea in naïve controls (Supplementary Video S3) and following induction of DED by desiccating stress (Supplementary Video S4). Representative IV-MPM still images of cDC migration in the limbus and cornea of naïve and DED mice are shown in Figure 3A. We observed that the track length of cDCs in the limbus was significantly increased in DED (80.73 ± 3.79 μm) compared to naïve mice (69.24 ± 2.53 μm, *p* = 0.033; Figures 3B,D). The difference was also present in the central cornea (115.60 ± 8.53 vs. 35.13 ± 5.60 μm, *p* < 0.001) and in the peripheral cornea (92.39 ± 6.85 vs. 59.51 ± 5.45 μm, *p* < 0.001; Figures 3C,D). Interestingly, we also noted a significantly longer displacement in cDCs in DED compared to naïve mice in the limbus (8.87 ± 0.68 vs. 6.63 ± 0.32 μm, *p* = 0.028) as well as central cornea (11.09 ± 1.68 vs. 4.59 ± 0.44 μm, *p* = 0.007). However, cDC displacement was comparable in the peripheral corneas of naïve (5.18 ± 0.44 μm) and DED mice (7.65 ± 1.33 μm, *p* = 0.080; Figure 3E).

Analysis of velocity revealed significant differences in 3D instantaneous velocity of ocular surface cDCs in DED compared to the naïve setting (Figure 3F). This increase was noted in cDCs within the limbus (1.70 ± 0.02 vs. 1.52 ± 0.01 μm/min, *p* < 0.001), peripheral (1.49 ± 0.02 vs. 1.35 ± 0.02 μm/min, *p* < 0.001), and central cornea (2.02 ± 0.04 vs. 1.14 ± 0.03 μm/min, *p* < 0.001) compared to naïve. Similarly, we observed a greater mean track speed of cDCs in DED compared to naïve mice in the limbus (2.69 ± 0.11 vs. 2.35 ± 0.05 μm/min, *p* = 0.036), peripheral (3.04 ± 0.18 vs. 2.12 ± 0.09 μm/min, *p* < 0.001), and central cornea (3.24 ± 0.20 vs. 1.60 ± 0.14 μm/min, *p* < 0.001; Figure 3G). Thus, our findings show that, while in naïve mice cDCs show minor motility, following exposure to desiccating stress, their kinetic features are altered, exhibiting higher kinesis with a faster speed.

To further assess the directionality of the movements of cDCs in the ocular surface, we measured meandering indices of cDCs in the limbus and cornea during DED, since during the steady state, the motility is limited. We noted a significant regional difference in the meandering index of limbal cDCs (0.30 ± 0.03) compared with cDCs in the central cornea (0.17 ± 0.04, *p* = 0.044; Figure 3H) in mice with DED. However, we did not observe a significant difference between peripheral (0.20 ± 0.03) and central corneal cDCs (*p* = 0.979; Figure 3H).

We also evaluated the regional differences in the kinetics of cDCs in the limbus, peripheral, and central cornea. We observed that in naïve mice, cDCs in the limbus traveled over longer tracks compared with cDCs in the central cornea (*p* = 0.008; Supplementary Figure S2D). However, in mice with DED, cDCs in the central cornea moved over longer tracks compared with cDCs in the limbus (*p* < 0.001; Supplementary Figure S2D). No significant differences were observed in the displacement of cDCs in the limbus, peripheral, and central cornea (Supplementary Figure S2E). In naïve mice, cDCs in the limbus showed higher 3D instantaneous velocity compared with cDCs in the peripheral (*p* < 0.001; Supplementary Figure S2F) and central cornea (*p* < 0.001; Supplementary Figure S2F). Furthermore, peripheral corneal cDCs migrated with a higher 3D instantaneous velocity compared with cDCs in the central cornea (*p* = 0.007; Supplementary Figure S2F). In mice with DED, cDCs in the central cornea exhibited higher 3D instantaneous velocity compared with cDCs in the limbus (*p* < 0.001; Supplementary Figure S2F) and peripheral cornea (*p* < 0.001; Supplementary Figure S2F). However, we did not detect significant differences in mean track speed of cDCs in the limbus, peripheral, and central corneas (Supplementary Figure S2G). In summary, we observed that during DED, cDCs exhibited altered migratory kinetics, with the highest pace in the peripheral and central cornea.

### The Impact of Nerve Contact on Alterations in Morphology and Kinetics of cDCs

**FIGURE 4:**
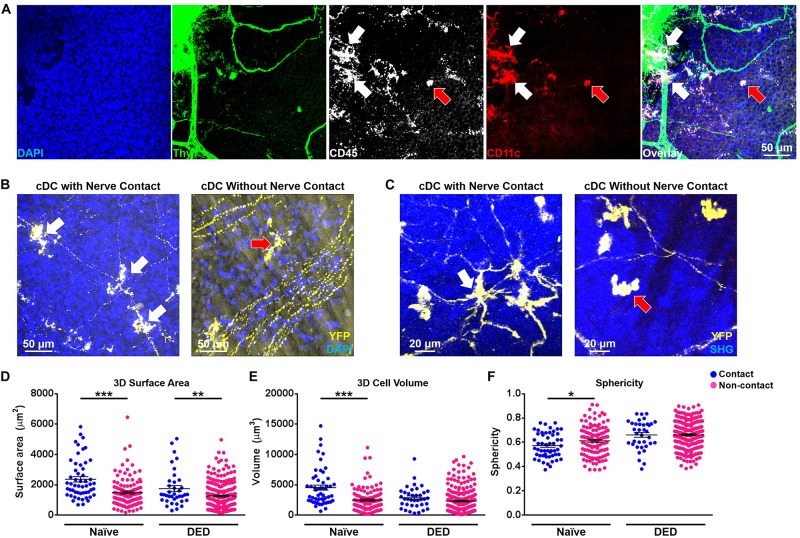
Morphological features of conventional dendritic cells based on their relation with nerves. **(A)** Representative confocal micrograph of corneal/limbal whole mount of a naïve wild-type mouse stained with Thy1, CD45, CD11c, and 4′,6-diamidino-2-phenylindole (DAPI) showing CD45^+^ CD11c^+^ conventional dendritic cells (cDCs) in contact (white arrows) and without contact (red arrow) with Thy1^+^ nerves. **(B)** Representative confocal micrograph of corneal/limbal whole-mount of a naïve CD11c^YFP^ × Thy1^YFP^ mouse mounted with DAPI showing YFP^+^ cDCs in contact (white arrows) and without contact (red arrow) with YFP^+^ nerves. **(C)** Representative intravital multiphoton microscopy (IV-MPM) images of peripheral corneal cDCs in contact (left panel, white arrow) and without contact (right panel, red arrow) with nerves in CD11c^YFP^ × Thy1^YFP^ mice after exposure to desiccating stress-induced dry eye disease (DED). **(D–F)** Morphological characteristics including **(D)** 3D cell surface area, **(E)** 3D cell volume, and **(F)** sphericity of cDCs in contact and without contact with nerves, pooled from at least three mice/group. Scale bars: 50 μm in **(A,B)** and 20 μm in **(C)**. Results are presented as mean ± SEM, *t* test. SHG, Second harmonic generation. *p < 0.05, **p < 0.01, and ***p < 0.001.

Associations between nerves and immune cells at the ocular surface have recently been reported (12, 44). Desiccating stress-induced DED has been noted to lead to morphological and functional ocular surface nerve alterations (25–28). Accordingly, we sought to investigate if the morphology and kinetic properties of cDCs in the ocular surface change when they were in contact with nerves in both DED and the naïve setting. In order to assess if we can visualize cDCs with and without contact with nerves, we initially performed confocal microscopy on whole-mounted limbus/cornea of wild-type mice during steady state. As presented in Figure 4A, we could detect cDCs, judged by expression of CD45 and CD11c, in contact with Thy1-expressing nerves (white arrows) as well as without contact with Thy1^+^ nerves (red arrow). Noteworthy, we observed that CD11c^+^ cells in contact (Supplementary Figure S3, white arrows) and without contact with nerves (Supplementary Figure S3, red arrows) do not costain with F4/80, confirming their identity as cDCs. Next, to assess if we can observe cDCs in contact with nerves in the limbus and cornea of CD11c^YFP^ × Thy1^YFP^ mice, we performed confocal microscopy on fresh corneal samples of double-transgenic naïve mice. As represented in Figure 4B, we identified cDCs, which were with contact (Figure 4B and Supplementary Figures S4A,B, white arrows; Supplementary Video S5) or without contact (Figure 4B and Supplementary Figures S4C,D, red arrows; Supplementary Video S6) with YFP^+^ nerves. Notably, in confocal micrographs, cDCs could be differentiated from Thy1^+^ nerves based on their morphology and presence of DAPI-stained nucleus (Supplementary Figures S4B,D; arrows). Next, we analyzed the frequency of cDCs with nerve contact (Figure 4C, white arrow; Supplementary Video S7) and without nerve contact (Figure 4C, red arrow; Supplementary Video S8) in naïve and DED mice via IV-MPM. We observed that, while 78.1 ± 3.6% of cDCs in the limbus were in contact with nerves during steady state, in mice with DED, only 47.9 ± 6.3% of cDCs in the limbus were in contact with nerves (*p* = 0.013; *n* = 3–5/group; Supplementary Figure S5). Notably, we observed a comparable reduction in the frequency of cDCs in contact with nerves in mice with DED in the peripheral and central cornea in mice with DED compared with naïve mice (*p* = 0.026 and *p* < 0.001, respectively; Supplementary Figure S5). Next, we analyzed 3D morphological parameters of cDCs in with and without nerve contact in naïve and DED mice using IV-MPM. Our analysis revealed that in naïve mice, cDCs in contact with nerves were significantly larger in terms of 3D cell surface area (2,359.0 ± 177.7 vs. 1,481.7 ± 90.0 μm^2^, *p* < 0.001; Figure 4D) and volume (4,522.9 ± 422.8 vs. 2,466.0 ± 175.7 μm^3^, *p* < 0.001; Figure 4E), but less spherical (0.57 ± 0.01 vs. 0.61 ± 0.01, *p* = 0.043; Figure 4F), compared to cDCs that are not in contact with nerves. Similarly, in the DED group, cDCs that were in contact with nerves had a greater 3D cell surface area compared to those not in contact with nerves (1,747.2 ± 193.9 vs. 1,255.0 ± 60.7 μm^2^, *p* = 0.004; Figure 4D). However, there were no significant differences in volume and sphericity of cDCs with and without nerve contact in DED (volume, 2,860.4 ± 293.7 vs. 2,308.9 ± 138.1 μm^3^, *p* = 0.129; sphericity, 0.66 ± 0.02 vs. 0.66 ± 0.01, *p* = 0.985, respectively; Figures 4E,F).

**FIGURE 5:**
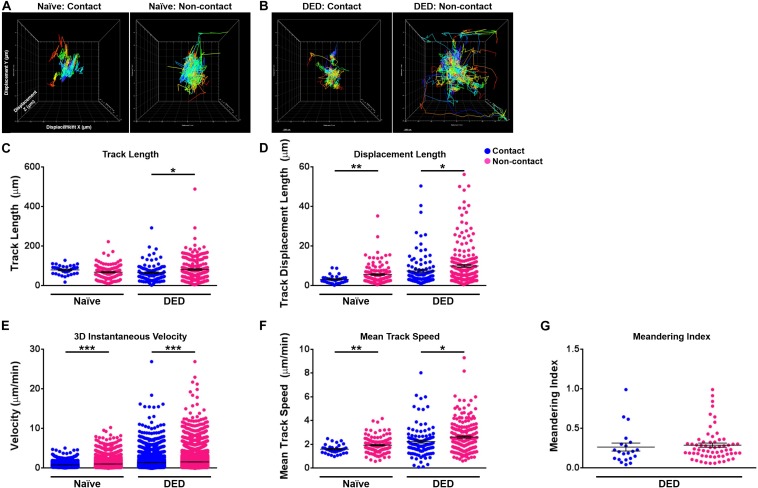
Kinetics of conventional dendritic cells according to their relation with nerves. **(A,B)** Representative displacement tracks of YFP^+^ conventional dendritic cells (cDCs) with and without nerve contact in **(A)** naïve and **(B)** dry eye disease (DED) mice with centered alignment shows reduced alterations in cDCs with nerve contact. **(C–G)** Kinetic characteristics including **(C)** track length, **(D)** displacement length, **(E)** 3D instantaneous velocity, **(F)** mean track speed, and **(G)** meandering indices of YFP^+^ cDC tracks in contact or not in contact with nerves, pooled from at least three mice/group. Results are presented as mean ± SEM, *t* test, *p < 0.05, **p < 0.01, and ***p < 0.001.

We next sought to investigate the effect of contact with nerves on cDC kinetics. As depicted in Figures 5A,B, we observed that cDCs in contact with nerves showed less motility in comparison with cDCs without apparent contact with nerves in both naïve (Figure 5A) and DED mice (Figure 5B). There were no significant differences in the track length (79.99 ± 4.20 vs. 68.41 ± 3.62 μm, *p* = 0.102; Figure 5C) of cDCs with and without nerve contact in the naïve setting. However, cDCs that are in contact with nerves showed a lower displacement (3.15 ± 0.36 vs. 5.62 ± 0.43 μm, *p* = 0.004; Figure 5D), 3D instantaneous velocity (0.83 ± 0.01 vs. 1.05 ± 0.01 μm/min, *p* < 0.001; Figure 5E), and mean track speed (1.60 ± 0.07 vs. 1.93 ± 0.06 μm/min, *p* = 0.005; Figure 5F) compared to cDCs not in contact with nerves. Interestingly, cDCs in contact with nerves showed a shorter track length (65.57 ± 4.24 vs. 81.64 ± 3.99 μm, *p* = 0.012; Figure 5C), less displacement (7.46 ± 0.78 vs. 9.96 ± 0.73 μm, *p* = 0.030; Figure 5D), lower 3D instantaneous velocity (1.39 ± 0.02 vs. 1.56 ± 0.01 μm/min, *p* < 0.001; Figure 5E), and mean track speed (2.24 ± 0.13 vs. 2.61 ± 0.09 μm/min, *p* = 0.017; Figure 5F) compared to cDCs not in contact with nerves in the DED setting. Finally, analysis of meandering index for cDCs in mice with DED revealed no significant difference in directionality between cDCs in contact and those not in contact with nerves (0.26 ± 0.05 vs. 0.29 ± 0.03, *p* = 0.65; Figure 5G). Thus, our results suggest that contact with nerves alters the aforementioned changes in the morphological properties and kinetics of cDCs in DED.

## Discussion

Through the use of IV-MPM on double-transgenic mice, we presented detailed alterations in the 3D morphology and kinetics of ocular surface cDCs following desiccating stress-induced DED and cDC-nerves interplay in this process. Multiple properties of IV-MPM, including its non-invasive nature, small focal point, second harmonic generation, and limited phototoxicity allow for real-time assessment of single cDC dynamics with high spatial and temporal resolution *in vivo*, without requiring antibodies and dyes for visualization. Using the strengths of IV-MPM, we showed that following exposure to desiccating stress, cDCs are increased in the ocular surface and exhibit remarkable alterations in their morphology and migratory characteristics. In fact, while during the steady state cDCs possess an elongated shape with long dendrites and are relatively static, they adopt a more migratory state (45) in DED, with less surface area and volume and more sphericity and migratory kinetics. Furthermore, we revealed that during DED, a significant fraction of cDCs lose their contact with nerves, which significantly alters their kinetics and 3D morphological parameters, suggesting the importance of neuroimmune interplay in the course of DED.

Several murine models have been developed to investigate DED. These include a wide range of genetic modifications, surgical techniques, and medical interventions to decrease tear production or secretion. Among less invasive approaches, inducing lacrimal gland insufficiency and atrophy by administrating agents such as parasympatholytic molecule scopolamine or imposing desiccating stress on the ocular surface by placing laboratory animals in controlled environment with low humidity and high flow rate are commonly used (37, 46–48). In order to avoid potential effects of surgical procedures, genetic modifications, and systemic medications on immune cells as well as potential off-target systemic effects of such manipulations, in this study, we aimed to induce DED solely by exposing the mice to environmental desiccating stress via a controlled environment chamber, as a model of desiccation-induced DED (37).

During steady state, cDCs reside among the basal layers of the epithelium as well as in the anterior stroma in the cornea (7, 49–51). We previously reported that during steady state, populations of corneal immune cells including cDCs are mainly static, only exhibiting centroid movement with minor displacement, suggestive of environmental sampling (41). In line with the prior report, we observed that cDCs are less motile in the naïve setting; however, greater track length, 3D instantaneous velocity, and mean track speed reaching ∼3 μm/min in the cornea are noted in cDCs following DED. Although there is paucity of similar studies in the literature to directly compare our findings, in a report investigating migration in the peripheral cornea and limbus following intrastromal suture-induced inflammation, auto-fluorescent immune cells were noted to have a mean speed of 9.5 μm/min, with a maximum speed of 54.0 μm/min (52). We previously reported that after thermal cautery burn, CD11c^+^ and MHC-II^+^ APCs have a mean speed of approximately 1.9 and 3.0 μm/min in the cornea, respectively (41). The differences between reports might be explained by the differences in the type of examined cells and the nature of inflammatory response in the cornea since, herein, we investigated DED as a subacute to chronic inflammatory state, while previous reports assessed acute corneal inflammation (41). Furthermore, we noted regional differences in the morphology and kinetics of cDCs. We observed that, in particular during DED, cDCs in the limbus harbor less surface area and volume and travel with lower 3D instantaneous velocity compared to central corneal cDCs. This maybe at least in part explained by some cDCs, which undergo morphological alterations that facilitate adhesion and extravasation from blood vessels or egress to draining lymph nodes in the limbal area.

Resident immune cells in the cornea are considered long-lived, since even after 6 months following transfer of bone marrow cells or bone marrow-derived hematopoietic stem cells to irradiated mice, not all immune cells in the cornea are replaced (53, 54). Transferred cells initially reach the limbus and later migrate to peripheral and subsequently to central corneas (53, 54); however, it is not yet clear if during the steady state, the resident immune cells of the cornea slowly self-regenerate through mitosis, arise from tissue resident precursors, or are recruited from the circulating blood (49, 53, 54). Nevertheless, upon inflammatory stimuli and increase in chemokines/cytokines in the cornea, cDCs are increased in the cornea, at least in part through recruitment from the blood (50, 51, 55). In this study, we observed that cDCs displayed longer track lengths in DED in the limbus, and exhibited a higher meandering index in the limbus compared with the corneal areas; thus, in line with prior studies, it might be postulated that cDCs recruited from the blood stream enter the tissue in the limbus, where they migrate to the peripheral and then the central cornea due to chemotactic gradients in the cornea during DED. However, in the cornea, cDCs did not show a preferential movement direction judged by centered displacement plots and meandering indices. This finding might be explained by the fact that desiccating stress, as the stimulus for inflammation, affects the cornea relatively evenly; thus, cDCs were not directed toward a specific location in the cornea. Additional investigation of immune cell kinetics at various time points following exposure to desiccating stress is required to elucidate a correlation between kinetics of cDCs and DED progression.

Association of immune cells and nerves has been reported in various peripheral and lymphoid tissues (56–61). In the cornea, the most densely innervated tissue of the body, cDCs and macrophages have been shown to reside in close proximity to the nerves in both humans and rodents (12, 44, 62–65). Although, during steady state, the immune cells display a close association with nerves in the cornea, it is shown that macrophages dissociate from nerves following corneal insults (12). Although the impact of this dissociation on the initiation or propagation of immune response, and the mechanisms associated with this warrant further in depth studies, it may be postulated that, upon receiving a danger signal directly from the microenvironment or through nerves, immune cells dissociate from nerves to freely surveil the tissue, take up antigens, and transfer them to draining lymph nodes. Secretory factors, such as cytokines, chemokines, and neurotransmitters, may play a role in the dissociation of immune cells from the nerves. In this regard, it has been shown that neurotransmitters, such as beta-endorphin, which are released by immune cells, may decrease the association of immune cells with dorsal nerve ganglion cells *in vitro* (66). In line with these observations, we demonstrate that cDCs in contact with nerves were larger, less spherical, and less motile compared to cDCs not in contact with nerves in naïve mice. However, although cDCs generally became smaller, more spherical, and motile in DED, cDCs in contact with nerves were still larger and less motile than other cDCs. These observations suggest that preservation of contact with nerves may prevent the morphological and kinetic alterations of cDCs in DED.

There is a growing body of evidence suggesting that the nervous system contributes to the control of local inflammation. Various studies have found that neurotransmitters and neuropeptides, such as substance P (SP), calcitonin gene-related peptide (CGRP), vasoactive intestinal polypeptide, secretoneurin, nerve-growth factor (NGF), and neuropeptide Y, are able to directly modulate the immune response in peripheral tissues (67–74). In this regard, the release of two key neuropeptides, CGRP and SP, by sensory nerves induces neurogenic inflammation (61, 75). These neuropeptides directly act on vascular endothelial and smooth muscle cells to enable vasodilation, increase capillary permeability, and stimulate leukocyte extravasation (76, 77) and thus play an important role in progression of DED (23, 78, 79). Despite the progress on neural control of inflammation, previous studies have focused on non-contact-mediated mechanisms through which nerves may regulate immune cells, whereas our study suggests the potential presence of additional contact-mediated mechanisms for neuroimmune interactions, since we observed differences in multiple parameters between cDCs with and without nerve contact. Notably, while cDC contact with nerves dampened cDC motility, it did not reduce cDC movement to naïve levels. This may be, in part, explained by the potential damage or dysfunction of nerves at the ocular surface as a result of DED or by the inflammatory microenvironment, impacting the immunomodulatory effect that nerves may have on cDCs.

It should also be noted that the corneal epithelial cells that provide support to the terminal nerve fibers extending toward the ocular surface (80) may also influence cDC–nerve association (44). Furthermore, it has previously been shown that morphology, thickness, and migration patterns of corneal epithelial cells may be altered in various corneal conditions (81–83). In particular, DED is associated with increased corneal epithelial cell turnover and thickness (84), likely due to an increase in the proliferation and migration of limbal epithelial stem cells via a natural centripetal migratory movement as a result of increased desquamation in DED (81, 85, 86). Therefore, the investigation of neuro-immune–epithelial interaction in steady state and disease states, such as in DED, may provide crucial information as to the impact of epithelial cells on neuro-immune crosstalk or vice versa. Considering that the prevalence of DED increases with age (3) and, conversely, corneal nerve density and sensitivity is decreased during aging (28), another avenue for future studies is to investigate the effect of aging on trafficking of immune cells and on neuro-immune interactions in the cornea.

Considering that the cornea is an avascular tissue, systemic administration of large molecules, such as antibodies, does not allow labeling of the corneal structures (87–89). Similarly, subconjunctival injection of antibodies generally does not lead to proper labeling of all corneal structures, such as the epithelium (90), in which a subset of immune cells reside. Moreover, intact corneal epithelium, as the most anterior layer of the cornea, does not allow for penetration of large molecules (90–92). Thus, to assess cDCs and nerves, in this study, we took benefit of transgenic mice with fluorescently labeled cDCs and nerves. However, this approach harbors several shortcomings. One of the limitations is that not all ocular surface nerves express Thy1 and hence YFP; thereby, we may have underestimated the number of cDCs in contact with nerves and falsely classified them to the non-contact group, which may have decreased the power of our comparisons. While this may indeed be the case, our results demonstrate significant differences in morphology and kinetics between cells in contact and not in contact with nerves. Furthermore, we determined the contact between corneal nerves and cDCs based on fluorescence signal detected by IV-MPM using Imaris, which enabled us to assess the spatial relation of each cell with surrounding nerves in 3D. Nevertheless, fluorescence signal may not reflect true contact. Therefore, future studies, utilizing alternative methodologies, such as transmission electron microscopy or *in vitro* co-cultures, are needed to confirm our findings. Another potential limitation of our study is the complicated process of preparing the mice for imaging, including application of local anesthetic drops, lubricant, and suture placement around the eye, all of which may potentially affect corneal nerve function and immune cell behavior. However, in this study, our control naïve mice also underwent a similar procedure for imaging, which limits potential inaccuracy of our findings. The presence of Thy1-YFP^+^ myeloid-derived cells, capable of secreting NGF following inflammation within the cornea, has previously been reported (93, 94). To address possible limitation of inclusion of these cells among the YFP^+^ cells in our CD11c^YFP^ × Thy1^YFP^ double-transgenic mice, we performed flow cytometric analysis on YFP^+^ cells and found that the majority of them align with a cDC phenotype. Therefore, the inclusion of Thy1-YFP^+^ cells in our analyses is likely negligible. Furthermore, in our transgenic CD11c^YFP^ × Thy1^YFP^ mice, both cDCs and nerves are labeled with a similar fluorescent protein. Although we could differentiate the YFP^+^ cDCs from YFP^+^ neuronal axons in the limbus and the cornea based on their morphology and presence/lack of movement, the application of transgenic mice with differential labeling of cDCs and nerves or alternative approaches to co-label cDCs or corneal nerves would be necessary to further elucidate fine alterations in the morphology of cDCs and nerves and studying potential membrane exchange between these structures in future studies. Nevertheless, we hope that the current study will stimulate further studies in this area.

Further studies are warranted to assess if removal from a desiccating stress environment after development of DED can lead to restoration of morphological and kinetic alterations of cDCs to the naïve setting, and if this is the case, how long is needed for such a recovery. Moreover, additional studies into the mechanisms of neuro-immune interactions at the ocular surface in disease and naïve states are warranted to categorically demonstrate the immunomodulatory role of ocular surface nerves as indicated by the novel intravital kinetic data presented herein. In summary, in this study, using IV-MPM, we illustrated that cDCs undergo morphological alterations and display increased migratory characteristics during DED that is, in part, ameliorated by corneal nerves. Taken together, we present *in vivo* evidence indicating that sensory nerves of the ocular surface may play an important role in modulating ocular surface immune cells in health and disease.

## Data Availability Statement

The datasets generated for this study are available on request to the corresponding author.

## Ethics Statement

The animal study was reviewed and approved by Tufts University, Tufts Medical Center Institutional Animal Care and Use Committee (IACUC).

## Author Contributions

AJ, YS-R, and PH designed the research. AJ, YS-R, CC, GO, BK, TB, and DH performed the research. AJ, YS-R, CC, GO, TB, DH, and PH analyzed the data. AJ, YS-R, CC, GO, BK, TB, DH, and PH prepared the manuscript.

## Conflict of Interest

The authors declare that the research was conducted in the absence of any commercial or financial relationships that could be construed as a potential conflict of interest.

## Supplementary Material

FIGURE S1 Flow cytometric characterization of YFP^+^ cells in CD11c^YFP^ × Thy1^YFP^ mice. **(A–C)** Sequential gating strategy to select live single YFP^+^ cells among bone marrow cell suspension of a naïve CD11c^YFP^ × Thy1^YFP^ mouse. **(A)** Gating out dead cells and debris and **(B)** doublets, and **(C)** gating on YFP^+^ cells. **(D)** Fluorescent minus one histograms for CD45, CD11b, CX3CR1, CD11b, PDCA-1, and CD68 on YFP^+^ cells. The experiment was repeated 3 times.

FIGURE S2 Regional differences in the morphology and kinetics of conventional dendritic cells (cDCs) in the limbus, peripheral, and central cornea. **(A–C)** Morphologic characteristics including 3D cell surface area **(A)**, 3D cell volume **(B)**, and sphericity **(C)** of YFP^+^ cDCs in naïve and DED conditions. **(D–G)** Kinetic characteristics including track length **(D)**, displacement length **(E)**, 3D instantaneous velocity **(F)**, and mean track speed **(G)** of YFP^+^ cDCs in transgenic CD11c^YFP^ × Thy1^YFP^ mice in naïve and DED conditions. Data is pooled from at least 3 mice/group. Results are presented as mean ± SEM, ANOVA with Tukey *post hoc*, ^∗^*p* < 0.05, ^∗∗^*p* < 0.01, and ^∗∗∗^*p* < 0.001.

FIGURE S3 Conventional dendritic cells in the cornea reside with or without contact with nerves. Representative confocal micrograph of corneal/limbal whole-mount of a naïve wild-type mouse stained with Thy1, CD11c, F4/80, and DAPI showing CD11c^+^ F4/80^*n**e**g*^ conventional dendritic cells can be found in contact (white arrows) or without contact (red arrows) with Thy1^+^ nerves. The experiment was repeated 3 times. Scale bar: 50 μm.

FIGURE S4 Morphologic differentiation of conventional dendritic cells and nerves in the cornea of transgenic CD11c^YFP^ × Thy1^YFP^ mice. Representative confocal micrograph of freshly excised corneal/limbal sample from a naïve CD11c^YFP^ × Thy1^YFP^ mouse mounted with DAPI. conventional dendritic cells (cDCs) exhibit a distinct morphology compared with nerves. **(A)** Representative maximum intensity projection of whole-mounted tissue, depicting cDCs in contact with nerves (white arrows). **(B)** Respective section on the level of nucleus shows presence of nucleus in the YFP^+^ cDCs (white arrows). **(C)** Representative maximum intensity projection of whole-mounted tissue, illustrating cDCs without contact with nerves (red arrow). **(D)** Respective section on the level of nucleus shows presence of nucleus in the YFP^+^ cDC (red arrow). The experiment was repeated 3 times. Scale bars: 50 μm.

FIGURE S5 Frequency of conventional dendritic cells in contact with nerves in the limbus and cornea of transgenic CD11c^YFP^ × Thy1^YFP^ mice. Quantification of relative frequency of YFP^+^ conventional dendritic cells in contact with nerves in the limbus, peripheral, and central cornea in naïve mice and following exposure to desiccating stress (*n* = 3–5/group). Results are presented as mean ± SEM, *t*-test, ^∗^*p* < 0.05, ^∗∗∗^*p* < 0.001.

VIDEO S1 Kinetics of conventional dendritic cells in the limbus of a naïve mouse. Representative intravital multi-photon microscopy movie of the limbus of a CD11c^YFP^ × Thy1^YFP^ transgenic mouse in naïve setting. As shown, conventional dendritic cells exhibited subtle locomotion over short tracks. Yellow: YFP, yellow fluorescent protein; Blue: SHG, second harmonic generation delineating collagen. White arrows points to cells with apparent movement. Scale bar: 50 μm.

VIDEO S2 Kinetics of conventional dendritic cells in the limbus of a mouse with dry eye disease. Representative intravital multi-photon microscopy movie of the limbus of a CD11c^YFP^ × Thy1^YFP^ transgenic mouse under exposure to desiccating stress. As shown, conventional dendritic cells displaced over longer tracks with a higher mean speed. Yellow: YFP, yellow fluorescent protein; Blue: SHG, second harmonic generation delineating collagen. White arrows points to cells with apparent movement. Scale bar: 50 μm.

VIDEO S3 Kinetics of conventional dendritic cells in the central cornea of a naïve mouse. Representative intravital multi-photon microscopy movie of the central cornea of a CD11c^YFP^ × Thy1^YFP^ transgenic mouse in naïve setting. As shown, conventional dendritic cells exhibited subtle locomotion over short tracks. Yellow; YFP, yellow fluorescent protein; Blue: SHG, second harmonic generation delineating collagen. Scale bar: 50 μm.

VIDEO S4 Kinetics of conventional dendritic cells in the central cornea of a mouse with dry eye disease. Representative intravital multi-photon microscopy movie of the central cornea of a CD11c^YFP^ × Thy1^YFP^ transgenic mouse under exposure to desiccating stress. As shown, conventional dendritic cells displaced over longer tracks with a higher mean speed. Yellow: YFP, yellow fluorescent protein; Blue: SHG, second harmonic generation delineating collagen. White arrows points to cells with apparent movement. Scale bar: 50 μm.

VIDEO S5 Confocal micrograph of conventional dendritic cells in contact with nerves. Representative 3D reconstructed video of confocal micrograph of a CD11c^YFP^ × Thy1^YFP^ transgenic mouse, indicating conventional dendritic cells (cDCs), containing nucleus, in contact with nerve. cDCs and nerves express YFP and are hence illustrated in yellow. Note the dendrite of the cDCs in contact with corneal nerves. Yellow: YFP, yellow fluorescent protein; Blue: DAPI, indicating nucleus. Scale bar: 50–60 μm.

VIDEO S6 Confocal micrograph of conventional dendritic cells without nerve contact. Representative 3D reconstructed video of confocal micrograph of a CD11c^YFP^ × Thy1^YFP^ transgenic mouse, indicating a conventional dendritic cell (cDC), containing nucleus, without nerve contact. cDCs and nerves express YFP and are hence illustrated in yellow. Note that the cDC is not in contact with corneal nerves. Yellow: YFP, yellow fluorescent protein; Blue: DAPI, indicating nucleus. Scale bar: 50 μm.

VIDEO S7 Intravital multiphoton microscopy ofconventional dendritic cells in contact with nerves. Representative intravital multi-photon microscopy image of a CD11c^YFP^ × Thy1^YFP^ transgenic mouse, indicating conventional dendritic cells (cDCs) in contact with nerve. cDCs and nerves express YFP and are hence illustrated in yellow. Note the dendrite of the cDC in contact with corneal nerves. Yellow; YFP, yellow fluorescent protein; Blue: SHG, second harmonic generation delineating collagen. Scale bar: 20 μm.

VIDEO S8 Intravital multiphoton microscopy of conventional dendritic cells without nerve contact. Representative intravital multi-photon microscopy image of a CD11c^YFP^ × Thy1^YFP^ transgenic mouse, indicating conventional dendritic cells (cDCs) without nerve contact. CDcs and nerves express YFP and are hence illustrated in yellow. Note that the cDC highlighted by the arrow is not in contact with corneal nerves. Yellow: YFP, yellow fluorescent protein; Blue: SHG, second harmonic generation delineating collagen. Scale bar: 20 μm.

## References

[1] Craig JP Nichols KK Akpek EK Caffery B Dua HS Joo CK TFOS DEWS II definition and classification report. *Ocul Surf.* (2017) 15:276–83. 10.1016/j.jtos.2017.05.008 28736335

[2] Stapleton F Alves M Bunya VY Jalbert I Lekhanont K Malet F TFOS DEWS II epidemiology report. *Ocul Surf.* (2017) 15:334–65. 10.1016/j.jtos.2017.05.003 28736337

[3] Dana R Bradley JL Guerin A Pivneva I Stillman IÖ Evans AM Estimated prevalence and incidence of Dry Eye disease based on coding analysis of a large, all-age United States health care system. *Am J Ophthalmol.* (2019) 202:47–54. 10.1016/j.ajo.2019.01.026 30721689

[4] Muller LJ Marfurt CF Kruse F Tervo TM Corneal nerves: structure, contents and function. *Exp Eye Res.* (2003) 76:521–42. 10.1016/s0014-4835(03)00050-2 12697417

[5] Al-Aqaba MA Fares U Suleman H Lowe J Dua HS Architecture and distribution of human corneal nerves. *Br J Ophthalmol.* (2010) 94:784–9. 10.1136/bjo.2009.173799 19889832

[6] Oliveira-Soto L Efron N Morphology of corneal nerves using confocal microscopy. *Cornea.* (2001) 20:374–84. 10.1046/j.1475-1313.2003.00106.x 11333324

[7] Hamrah P Zhang Q Liu Y Dana MR Novel characterization of MHC class II-negative population of resident corneal Langerhans cell-type dendritic cells. *Invest Ophthalmol Vis Sci.* (2002) 43:639–46. 11867578

[8] Brissette-Storkus CS Reynolds SM Lepisto AJ Hendricks RL Identification of a novel macrophage population in the normal mouse corneal stroma. *Invest Ophthalmol Vis Sci.* (2002) 43:2264–71. 12091426 PMC3253392

[9] Hamrah P Liu Y Zhang Q Dana MR The corneal stroma is endowed with a significant number of resident dendritic cells. *Invest Ophthalmol Vis Sci.* (2003) 44:581–9. 10.1167/iovs.02-0838 12556386

[10] Chinnery HR Ruitenberg MJ Plant GW Pearlman E Jung S McMenamin PG The chemokine receptor CX3CR1 mediates homing of MHC class II-positive cells to the normal mouse corneal epithelium. *Invest Ophthalmol Vis Sci.* (2007) 48:1568–74. 10.1167/iovs.06-0746 17389486 PMC3392181

[11] Hattori T Chauhan SK Lee H Ueno H Dana R Kaplan DH Characterization of Langerin-expressing dendritic cell subsets in the normal cornea. *Invest Ophthalmol Vis Sci.* (2011) 52:4598–604. 10.1167/iovs.10-6741 21482644 PMC3175952

[12] Seyed-Razavi Y Chinnery HR McMenamin P G. A novel association between resident tissue macrophages and nerves in the peripheral stroma of the murine cornea. *Invest Ophthalmol Vis Sci.* (2014) 55:1313–20. 10.1167/iovs.13-12995 24458151

[13] Johnson ME Murphy PJ Changes in the tear film and ocular surface from dry eye syndrome. *Prog Retin Eye Res.* (2004) 23:449–74. 10.1016/j.preteyeres.2004.04.003 15219877

[14] Calonge M Enríquez-de-Salamanca A Diebold Y González-García MJ Reinoso R Herreras JM Dry eye disease as an inflammatory disorder. *Ocul Immunol Inflamm.* (2010) 18:244–53. 10.3109/09273941003721926 20482396

[15] No authors listed. Research in dry eye: report of the research subcommittee of the International Dry Eye Workshop. (2007). *Ocul Surf.* (2007) 5:179–93. 10.1016/s1542-0124(12)70086-1 17508121

[16] Stern ME Schaumburg CS Pflugfelder SC Dry eye as a mucosal autoimmune disease. *Int Rev Immunol.* (2013) 32:19–41. 10.3109/08830185.2012.748052 23360156 PMC3587314

[17] Barabino S Chen Y Chauhan S Dana R Ocular surface immunity: homeostatic mechanisms and their disruption in dry eye disease. *Prog Retin Eye Res.* (2012) 31:271–85. 10.1016/j.preteyeres.2012.02.003 22426080 PMC3334398

[18] Gandhi NB Su Z Zhang X Volpe EA Pelegrino FS Rahman SA Dendritic cell-derived thrombospondin-1 is critical for the generation of the ocular surface Th17 response to desiccating stress. *J Leukoc Biol.* (2013) 94:1293–301. 10.1189/jlb.1012524 23983225 PMC4051277

[19] Pflugfelder SC Corrales RM de Paiva CS T helper cytokines in dry eye disease. *Exp Eye Res.* (2013) 117:118–25. 10.1016/j.exer.2013.08.013 24012834 PMC3855838

[20] De Paiva CS Villarreal AL Corrales RM Rahman HT Chang VY Farley WJ Dry eye-induced conjunctival epithelial squamous metaplasia is modulated by interferon-gamma. *Invest Ophthalmol Vis Sci.* (2007) 48: 2553–60. 10.1167/iovs.07-0069 17525184

[21] Rivas L Oroza MA Perez-Esteban A Murube-del-Castillo J Morphological changes in ocular surface in dry eyes and other disorders by impression cytology. *Graefes Arch Clin Exp Ophthalmol.* (1992) 230:329–34. 10.1007/bf00165940 1505763

[22] Belmonte C Aracil A Acosta MC Luna C Gallar J Nerves and sensations from the eye surface. *Ocul Surf.* (2004) 2:248–53. 10.1016/s1542-0124(12)70112-x 17216099

[23] Mantelli F Massaro-Giordano M Macchi I Lambiase A Bonini S The cellular mechanisms of dry eye: from pathogenesis to treatment. *J Cell Physiol.* (2013) 228:2253–6. 10.1002/jcp.24398 23696296

[24] Belmonte C Nichols JJ Cox SM Brock JA Begley CG Bereiter DA TFOS DEWS II pain and sensation report. *Ocul Surf.* (2017) 15:404–37. 10.1016/j.jtos.2017.05.002 28736339 PMC5706540

[25] Benitez del Castillo JM Wasfy MA Fernandez C Garcia-Sanchez J An in vivo confocal masked study on corneal epithelium and subbasal nerves in patients with dry eye. *Invest Ophthalmol Vis Sci.* (2004) 45:3030–5. 10.1167/iovs.04-0251 15326117

[26] Bourcier T Acosta MC Borderie V Borrás F Gallar J Bury T Decreased corneal sensitivity in patients with dry eye. *Invest Ophthalmol Vis Sci.* (2005) 46:2341–5. 10.1167/iovs.04-1426 15980220

[27] Simsek C Kojima T Dogru M Tsubota K Alterations of murine subbasal corneal nerves after environmental Dry Eye stress. *Invest Ophthalmol Vis Sci.* (2018) 59:1986–95. 10.1167/iovs.17-23743 29677361

[28] Stepp MA Pal-Ghosh S Tadvalkar G Williams A Pflugfelder SC de Paiva CS Reduced intraepithelial corneal nerve density and sensitivity accompany desiccating stress and aging in C57BL/6 mice. *Exp Eye Res.* (2018) 169:91–8. 10.1016/j.exer.2018.01.024 29407221 PMC5949876

[29] Dieckmann G Goyal S Hamrah P Neuropathic corneal pain: approaches for management. *Ophthalmology.* (2017) 124:S34–47. 10.1016/j.ophtha.2017.08.004 29055360 PMC5743225

[30] Galor A Moein HR Lee C Rodriguez A Felix ER Sarantopoulos KD Neuropathic pain and dry eye. *Ocul Surf.* (2018) 16:31–44. 10.1016/j.jtos.2017.10.001 29031645 PMC5756672

[31] Baudouin C Aragona P Messmer EM Tomlinson A Calonge M Boboridis KG Role of hyperosmolarity in the pathogenesis and management of dry eye disease: proceedings of the OCEAN group meeting. *Ocul Surf.* (2013) 11:246–58. 10.1016/j.jtos.2013.07.003 24112228

[32] Yagci A Gurdal C The role and treatment of inflammation in dry eye disease. *Int Ophthalmol.* (2014) 34:1291–301. 10.1007/s10792-014-9969-x 25416345

[33] Cruzat A Qazi Y Hamrah P In vivo confocal microscopy of corneal nerves in health and disease. *Ocul Surf.* (2017) 15:15–47. 10.1016/j.jtos.2016.09.004 27771327 PMC5512932

[34] McKay TB Seyed-Razavi Y Ghezzi CE Dieckmann G Nieland TJF Cairns DM Corneal pain and experimental model development. *Prog Retin Eye Res.* (2018) 71:88–113. 10.1016/j.preteyeres.2018.11.005 30453079 PMC6690397

[35] Lindquist RL Shakhar G Dudziak D Wardemann H Eisenreich T Dustin ML Visualizing dendritic cell networks in vivo. *Nat Immunol.* (2004) 5:1243–50. 10.1038/ni1139 15543150

[36] de Paiva CS Effects of aging in Dry Eye. *Int Ophthalmol Clin.* (2017) 57:47–64. 10.1097/iio.0000000000000170 28282314 PMC5347479

[37] Barabino S Shen L Chen L Rashid S Rolando M Dana MR The controlled-environment chamber: a new mouse model of dry eye. *Invest Ophthalmol Vis Sci.* (2005) 46:2766–71. 10.1167/iovs.04-1326 16043849

[38] Lemp MA Report of the National Eye Institute/industry workshop on clinical trials in Dry Eyes. *CLAO J.* (1995) 21:221–32. 8565190

[39] Kilic S Kulualp K Tear production rate in a mouse model of Dry Eye according to the phenol red thread and endodontic absorbent paper point tear tests. *Comp Med.* (2016) 66:367–72. 27780003 PMC5073061

[40] Dursun D Wang M Monroy D Li DQ Lokeshwar BL Stern ME A mouse model of keratoconjunctivitis sicca. *Invest Ophthalmol Vis Sci.* (2002) 43:632–8. 11867577

[41] Seyed-Razavi Y Lopez MJ Mantopoulos D Zheng L Massberg S Sendra VG Kinetics of corneal leukocytes by intravital multiphoton microscopy. *FASEB J.* (2019) 33:2199–211. 10.1096/fj.201800684RR 30226811 PMC6338630

[42] Jamali A Harris DL Blanco T Lopez MJ Hamrah P Resident plasmacytoid dendritic cells patrol vessels in the naïve limbus and conjunctiva. *Ocul Surf.* (2020) 18:277–85. 10.1016/j.jtos.2020.02.005 32109562 PMC7397780

[43] Sumen C Mempel TR Mazo IB von Andrian UH Intravital microscopy: visualizing immunity in context. *Immunity.* (2004) 21:315–29. 10.1016/j.immuni.2004.08.006 15357943

[44] Gao N Lee P Yu FS Intraepithelial dendritic cells and sensory nerves are structurally associated and functional interdependent in the cornea. *Sci Rep.* (2016) 6:36414. 10.1038/srep36414 27805041 PMC5090364

[45] Lammermann T Germain RN The multiple faces of leukocyte interstitial migration. *Semin Immunopathol.* (2014) 36:227–51. 10.1007/s00281-014-0418-8 24573488 PMC4118216

[46] Guzman M Keitelman I Sabbione F Trevani AS Giordano MN Galletti JG Desiccating stress-induced disruption of ocular surface immune tolerance drives dry eye disease. *Clin Exp Immunol.* (2016) 184:248–56. 10.1111/cei.12759 26690299 PMC4837231

[47] De Paiva CS Chotikavanich S Pangelinan SB Pitcher JD III Fang B Zheng X IL-17 disrupts corneal barrier following desiccating stress. *Mucosal Immunol.* (2009) 2:243–53. 10.1038/mi.2009.5 19242409 PMC3594767

[48] Barbosa FL Xiao Y Bian F Coursey TG Ko BY Clevers H Goblet cells contribute to ocular surface immune tolerance-implications for Dry Eye disease. *Int J Mol Sci.* (2017) 18:978. 10.3390/ijms18050978 28475124 PMC5454891

[49] Chen W Hara K Tian Q Zhao K Yoshitomi T Existence of small slow-cycling Langerhans cells in the limbal basal epithelium that express ABCG2. *Exp Eye Res.* (2007) 84:626–34. 10.1016/j.exer.2006.11.006 17254566

[50] Hamrah P Huq SO Liu Y Zhang Q Dana MR Corneal immunity is mediated by heterogeneous population of antigen-presenting cells. *J Leukoc Biol.* (2003) 74:172–8. 10.1189/jlb.1102544 12885933

[51] Hamrah P Liu Y Zhang Q Dana MR Alterations in corneal stromal dendritic cell phenotype and distribution in inflammation. *Arch Ophthalmol.* (2003) 121:1132–40. 10.1001/archopht.121.8.1132 12912691

[52] Steven P Bock F Huttmann G Cursiefen C Intravital two-photon microscopy of immune cell dynamics in corneal lymphatic vessels. *PLoS One.* (2011) 6:e26253. 10.1371/journal.pone.0026253 22028842 PMC3197633

[53] Chinnery HR Humphries T Clare A Dixon AE Howes K Moran CB Turnover of bone marrow-derived cells in the irradiated mouse cornea. *Immunology.* (2008) 125:541–8. 10.1111/j.1365-2567.2008.02868.x 18540963 PMC2612551

[54] Nakamura T Ishikawa F Sonoda KH Hisatomi T Qiao H Yamada J Characterization and distribution of bone marrow-derived cells in mouse cornea. *Invest Ophthalmol Vis Sci.* (2005) 46:497–503. 10.1167/iovs.04-1154 15671274

[55] Lopez MJ Seyed-Razavi Y Jamali A Harris DL Hamrah P The chemokine receptor CXCR4 mediates recruitment of CD11c+ conventional dendritic cells into the inflamed murine cornea. *Invest Ophthalmol Vis Sci.* (2018) 59:5671–81. 10.1167/iovs.18-25084 30489627 PMC6266730

[56] Veres TZ Shevchenko M Krasteva G Spies E Prenzler F Rochlitzer S Dendritic cell-nerve clusters are sites of T cell proliferation in allergic airway inflammation. *Am J Pathol.* (2009) 174:808–17. 10.2353/ajpath.2009.080800 19179611 PMC2665742

[57] Hu D Nicholls PK Claus M Wu Y Shi Z Greene WK Immunofluorescence characterization of innervation and nerve-immune cell interactions in mouse lymph nodes. *Eur J Histochem.* (2019) 63:3059. 10.4081/ejh.2019.3059 31631646 PMC6802453

[58] Al-Shalan HAM Hu D Nicholls PK Greene WK Ma B Immunofluorescent characterization of innervation and nerve-immune cell neighborhood in mouse thymus. *Cell Tissue Res.* (2019) 378:239–54. 10.1007/s00441-019-03052-4 31230166

[59] Veres TZ Rochlitzer S Shevchenko M Fuchs B Prenzler F Nassenstein C Spatial interactions between dendritic cells and sensory nerves in allergic airway inflammation. *Am J Respir Cell Mol Biol.* (2007) 37:553–61. 10.1165/rcmb.2007-0087OC 17600312

[60] Heuss ND Pierson MJ Montaniel KR McPherson SW Lehmann U Hussong SA Retinal dendritic cell recruitment, but not function, was inhibited in MyD88 and TRIF deficient mice. *J Neuroinflamm.* (2014) 11:143. 10.1186/s12974-014-0143-1 25116321 PMC4149240

[61] Chiu IM von Hehn CA Woolf CJ Neurogenic inflammation and the peripheral nervous system in host defense and immunopathology. *Nat Neurosci.* (2012) 15:1063–7. 10.1038/nn.3144 22837035 PMC3520068

[62] Patel DV McGhee CN Mapping of the normal human corneal sub-Basal nerve plexus by in vivo laser scanning confocal microscopy. *Invest Ophthalmol Vis Sci.* (2005) 46:4485–8. 10.1167/iovs.05-0794 16303938

[63] Zhivov A Stave J Vollmar B Guthoff R In vivo confocal microscopic evaluation of Langerhans cell density and distribution in the normal human corneal epithelium. *Graefes Arch Clin Exp Ophthalmol.* (2005) 243:1056–61. 10.1007/s00417-004-1075-8 15856272

[64] Cruzat A Witkin D Baniasadi N Zheng L Ciolino JB Jurkunas UV Inflammation and the nervous system: the connection in the cornea in patients with infectious keratitis. *Invest Ophthalmol Vis Sci.* (2011) 52:5136–43. 10.1167/iovs.10-7048 21460259 PMC3176064

[65] Leppin K Behrendt AK Reichard M Stachs O Guthoff RF Baltrusch S Diabetes mellitus leads to accumulation of dendritic cells and nerve fiber damage of the subbasal nerve plexus in the cornea. *Invest Ophthalmol Vis Sci.* (2014) 55:3603–15. 10.1167/iovs.14-14307 24781935

[66] Hua S Hermanussen S Tang L Monteith GR Cabot PJ The neural cell adhesion molecule antibody blocks cold water swim stress-induced analgesia and cell adhesion between lymphocytes and cultured dorsal root ganglion neurons. *Anesth Analg.* (2006) 103:1558–64. 10.1213/01.ane.0000243410.61451.c1 17122239

[67] Sasaoka A Ishimoto I Kuwayama Y Sakiyama T Manabe R Shiosaka S Overall distribution of substance P nerves in the rat cornea and their three-dimensional profiles. *Invest Ophthalmol Vis Sci.* (1984) 25:351–6. 6199322

[68] Dunzendorfer S Kaser A Meierhofer C Tilg H Wiedermann CJ Cutting edge: peripheral neuropeptides attract immature and arrest mature blood-derived dendritic cells. *J Immunol.* (2001) 166:2167–72. 10.4049/jimmunol.166.4.2167 11160268

[69] Seiffert K Granstein RD Neuroendocrine regulation of skin dendritic cells. *Ann N Y Acad Sci.* (2006) 1088:195–206. 10.1196/annals.1366.011 17192566

[70] Szliter EA Lighvani S Barrett RP Hazlett LD Vasoactive intestinal peptide balances pro- and anti-inflammatory cytokines in the *Pseudomonas aeruginosa*-infected cornea and protects against corneal perforation. *J Immunol.* (2007) 178:1105–14. 10.4049/jimmunol.178.2.1105 17202374

[71] Souza-Moreira L Campos-Salinas J Caro M Gonzalez-Rey E Neuropeptides as pleiotropic modulators of the immune response. *Neuroendocrinology.* (2011) 94:89–100. 10.1159/000328636 21734355

[72] Oh JY Choi H Lee RH Roddy GW Ylöstalo JH Wawrousek E Identification of the HSPB4/TLR2/NF-kappaB axis in macrophage as a therapeutic target for sterile inflammation of the cornea. *EMBO Mol Med.* (2012) 4:435–48. 10.1002/emmm.201200221 22359280 PMC3403300

[73] Lambrecht BN Immunologists getting nervous: neuropeptides, dendritic cells and T cell activation. *Respir Res.* (2001) 2:133–8. 10.1186/rr49 11686876 PMC2002076

[74] Troger J Kieselbach G Teuchner B Kralinger M Nguyen QA Haas G Peptidergic nerves in the eye, their source and potential pathophysiological relevance. *Brain Res Rev.* (2007) 53:39–62. 10.1016/j.brainresrev.2006.06.002 16872680

[75] Godinho-Silva C Cardoso F Veiga-Fernandes H Neuro-immune cell units: a new paradigm in physiology. *Ann Rev Immunol.* (2019) 37:19–46. 10.1146/annurev-immunol-042718-041812 30379595

[76] McCormack DG Mak JC Coupe MO Barnes PJ Calcitonin gene-related peptide vasodilation of human pulmonary vessels. *J Appl Physiol (1985).* (1989) 67:1265–70. 10.1152/jappl.1989.67.3.1265 2551879

[77] Springer J Geppetti P Fischer A Groneberg DA Calcitonin gene-related peptide as inflammatory mediator. *Pulm Pharmacol Ther.* (2003) 16:121–30. 10.1016/S1094-5539(03)00049-X 12749828

[78] Beuerman RW Stern ME Neurogenic inflammation: a first line of defense for the ocular surface. *Ocul Surf.* (2005) 3:S203–6. 10.1016/s1542-0124(12)70256-2 17216120

[79] Mantelli F Micera A Sacchetti M Bonini S Neurogenic inflammation of the ocular surface. *Curr Opin Allergy Clin Immunol.* (2010) 10:498–504. 10.1097/ACI.0b013e32833e16cc 20706114

[80] Stepp MA Tadvalkar G Hakh R Pal-Ghosh S Corneal epithelial cells function as surrogate Schwann cells for their sensory nerves. *Glia.* (2017) 65:851–63. 10.1002/glia.23102 27878997 PMC5395310

[81] Lemp MA Mathers WD Corneal epithelial cell movement in humans. *Eye (Lond).* (1989) 3(Pt 4):438–45. 10.1038/eye.1989.65 2606218

[82] Walczysko P Rajnicek AM Collinson JM Contact-mediated control of radial migration of corneal epithelial cells. *Mol Vis.* (2016) 22:990–1004. 27563231 PMC4976620

[83] Lu F Simpson T Sorbara L Fonn D Malleability of the ocular surface in response to mechanical stress induced by orthokeratology contact lenses. *Cornea.* (2008) 27:133–41. 10.1097/ICO.0b013e318158b4b5 18216565

[84] Fabiani C Barabino S Rashid S Dana MR Corneal epithelial proliferation and thickness in a mouse model of dry eye. *Exp Eye Res.* (2009) 89:166–71. 10.1016/j.exer.2009.03.003 19298814 PMC2713794

[85] Nagasaki T Zhao J Centripetal movement of corneal epithelial cells in the normal adult mouse. *Invest Ophthalmol Vis Sci.* (2003) 44:558–66. 10.1167/iovs.02-0705 12556383

[86] Di Girolamo N Bobba S Raviraj V Delic NC Slapetova I Nicovich PR Tracing the fate of limbal epithelial progenitor cells in the murine cornea. *Stem Cells.* (2015) 33:157–69. 10.1002/stem.1769 24966117

[87] Howes EL Cruse VK Kwok MT Mononuclear cells in the corneal response to endotoxin. *Invest Ophthalmol Vis Sci.* (1982) 22:494–501. 7037677

[88] BenEzra D Maftzir G Hochberg E Anteby I Lorberboum-Galski H Ocular distribution of the chimeric protein IL2-PE40. *Curr Eye Res.* (1995) 14:153–8. 10.3109/02713689508999927 7768107

[89] Kim YC Chiang B Wu X Prausnitz MR Ocular delivery of macromolecules. *J Control Release.* (2014) 190:172–81. 10.1016/j.jconrel.2014.06.043 24998941 PMC4142116

[90] Dastjerdi MH Sadrai Z Saban DR Zhang Q Dana R Corneal penetration of topical and subconjunctival bevacizumab. *Invest Ophthalmol Vis Sci.* (2011) 52:8718–23. 10.1167/iovs.11-7871 22003112 PMC3230312

[91] Thiel MA Coster DJ Standfield SD Brereton HM Mavrangelos C Zola H Penetration of engineered antibody fragments into the eye. *Clin Exp Immunol.* (2002) 128:67–74. 10.1046/j.1365-2249.2002.01808.x 11982592 PMC1906367

[92] Mantelli F Mauris J Argueso P The ocular surface epithelial barrier and other mechanisms of mucosal protection: from allergy to infectious diseases. *Curr Opin Allergy Clin Immunol.* (2013) 13:563–8. 10.1097/ACI.0b013e3283645899 23974687 PMC3858173

[93] Sarkar J Chaudhary S Namavari A Ozturk O Chang JH Yco L Corneal neurotoxicity due to topical benzalkonium chloride. *Invest Ophthalmol Vis Sci.* (2012) 53:1792–802. 10.1167/iovs.11-8775 22410563 PMC3995561

[94] Sarkar J Chaudhary S Jassim SH Ozturk O Chamon W Ganesh B CD11b+GR1+ myeloid cells secrete NGF and promote trigeminal ganglion neurite growth: implications for corneal nerve regeneration. *Invest Ophthalmol Vis Sci.* (2013) 54:5920–36. 10.1167/iovs.13-12237 23942970 PMC3762328

