# Supplementary figures and images for: Intravital Multiphoton Microscopy of the Ocular Surface: Alterations in Conventional Dendritic Cell Morphology and Kinetics in Dry Eye Disease
Source: Front Immunol. 2020 May 7;11:742. doi: 10.3389/fimmu.2020.00742 (PMC7227427; doi:10.3389/fimmu.2020.00742)

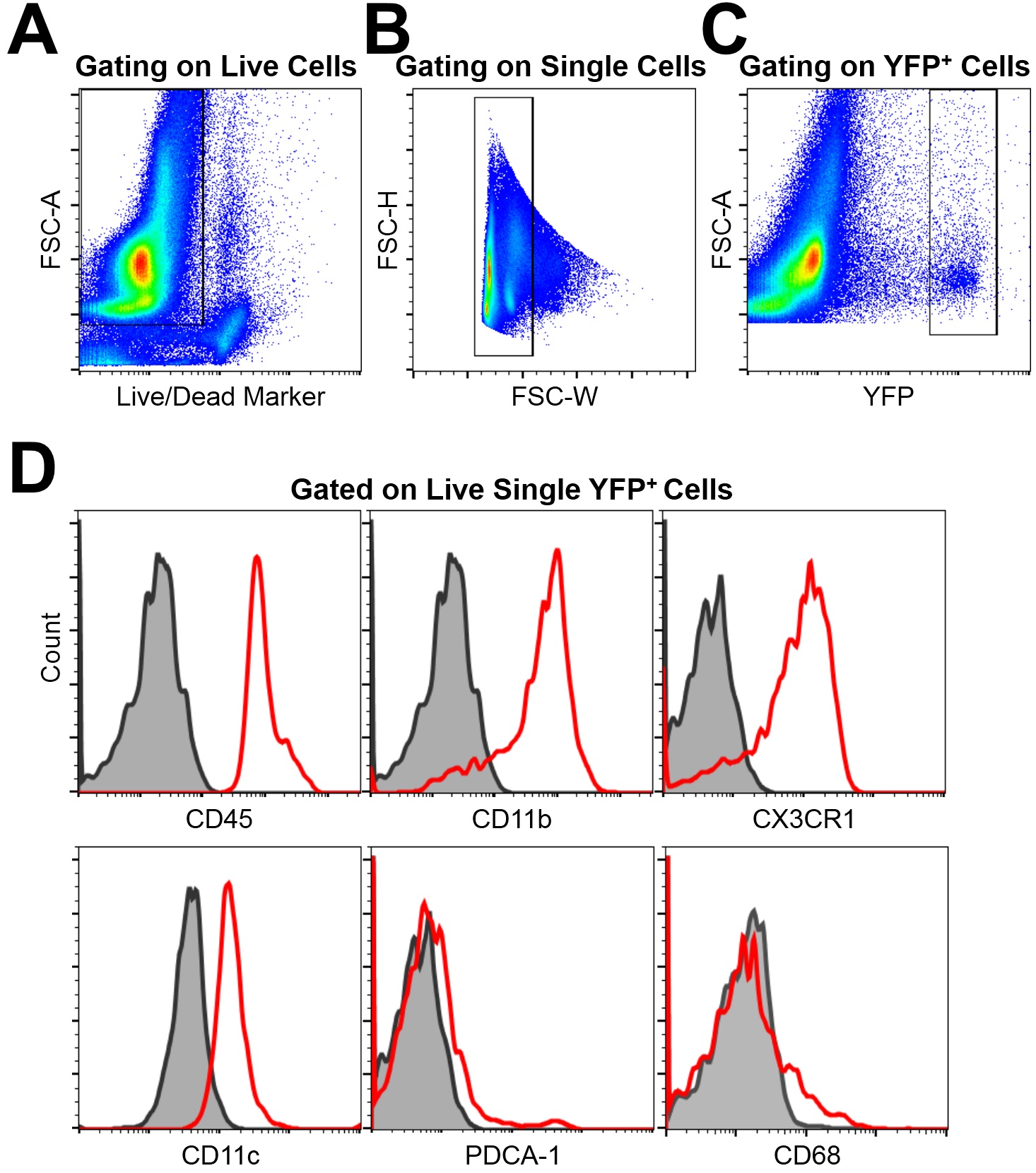

Supplement: FIGURE S1 — Flow cytometric characterization of YFP+ cells in CD11cYFP × Thy1YFP mice. (A–C) Sequential gating strategy to select live single YFP+ cells among bone marrow cell suspension of a naïve CD11cYFP × Thy1YFP mouse. (A) Gating out dead cells and debris and (B) doublets, and (C) gating on YFP+ cells. (D) Fluorescent minus one histograms for CD45, CD11b, CX3CR1, CD11b, PDCA-1, and CD68 on YFP+ cells. The experiment was repeated 3 times. [file Image_1.JPEG]

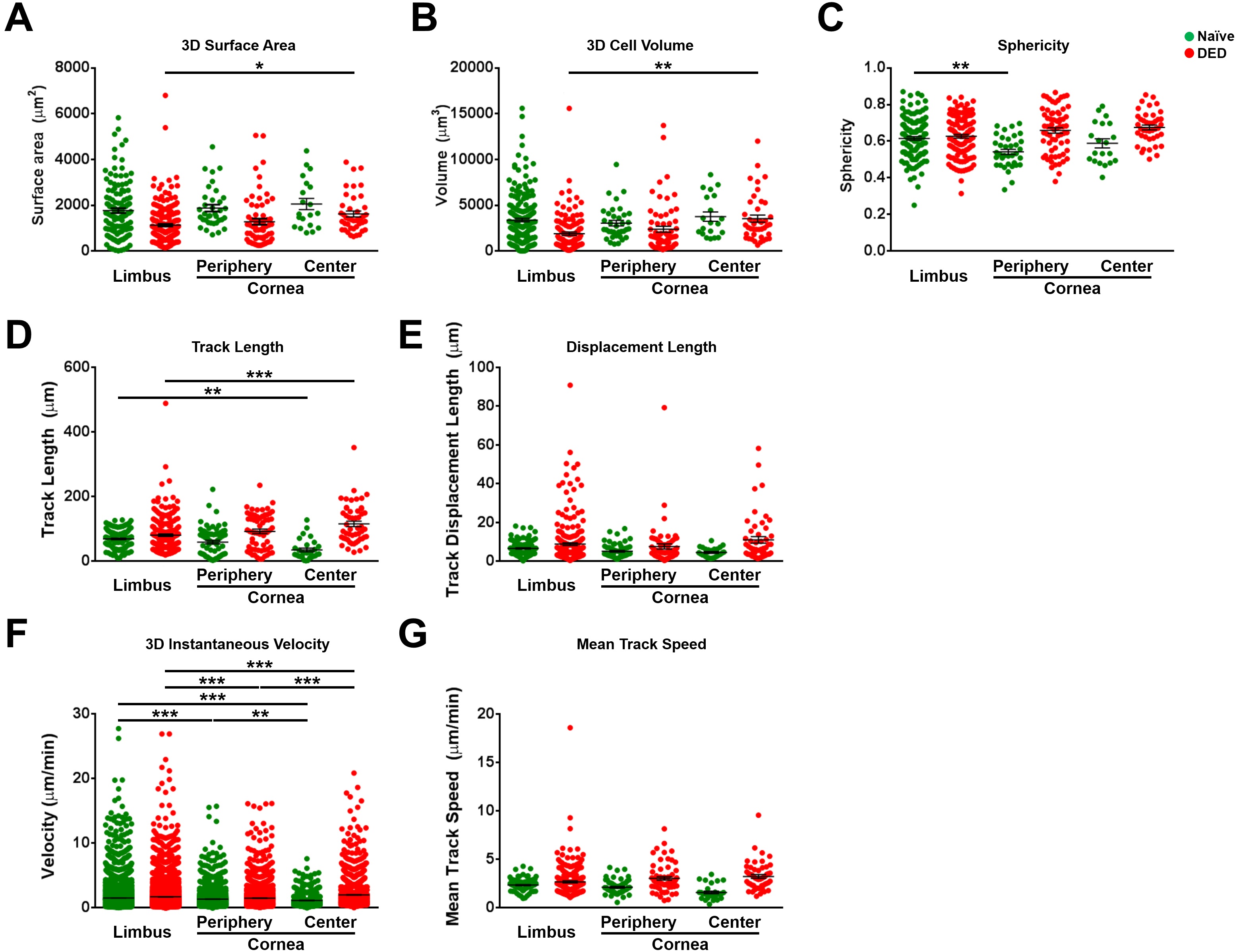

Supplement: FIGURE S2 — Regional differences in the morphology and kinetics of conventional dendritic cells (cDCs) in the limbus, peripheral, and central cornea. (A–C) Morphologic characteristics including 3D cell surface area (A), 3D cell volume (B), and sphericity (C) of YFP+ cDCs in naïve and DED conditions. (D–G) Kinetic characteristics including track length (D), displacement length (E), 3D instantaneous velocity (F), and mean track speed (G) of YFP+ cDCs in transgenic CD11cYFP × Thy1YFP mice in naïve and DED conditions. Data is pooled from at least 3 mice/group. Results are presented as mean ± SEM, ANOVA with Tukey post hoc, ∗p < 0.05, ∗∗p < 0.01, and ∗∗∗p < 0.001. [file Image_2.jpg]

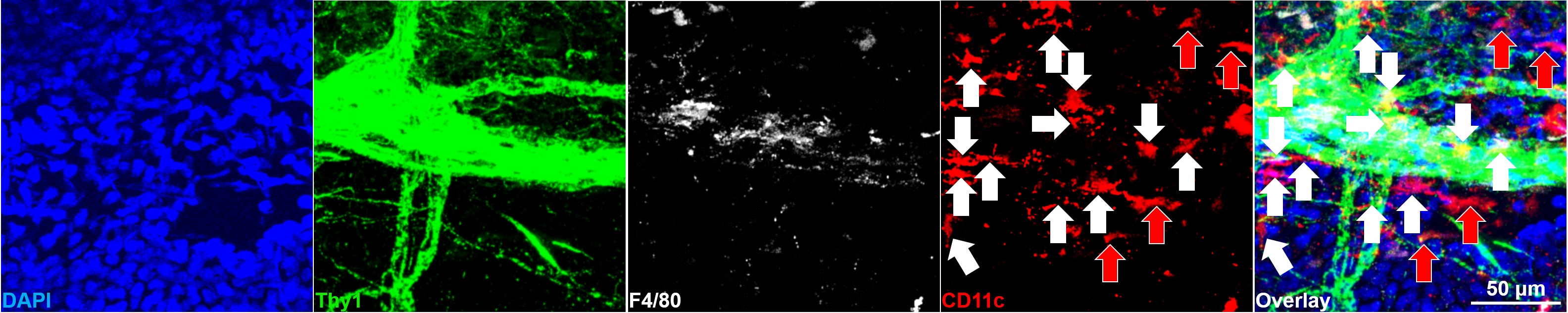

Supplement: FIGURE S3 — Conventional dendritic cells in the cornea reside with or without contact with nerves. Representative confocal micrograph of corneal/limbal whole-mount of a naïve wild-type mouse stained with Thy1, CD11c, F4/80, and DAPI showing CD11c+ F4/80neg conventional dendritic cells can be found in contact (white arrows) or without contact (red arrows) with Thy1+ nerves. The experiment was repeated 3 times. Scale bar: 50 μm. [file Image_3.JPEG]

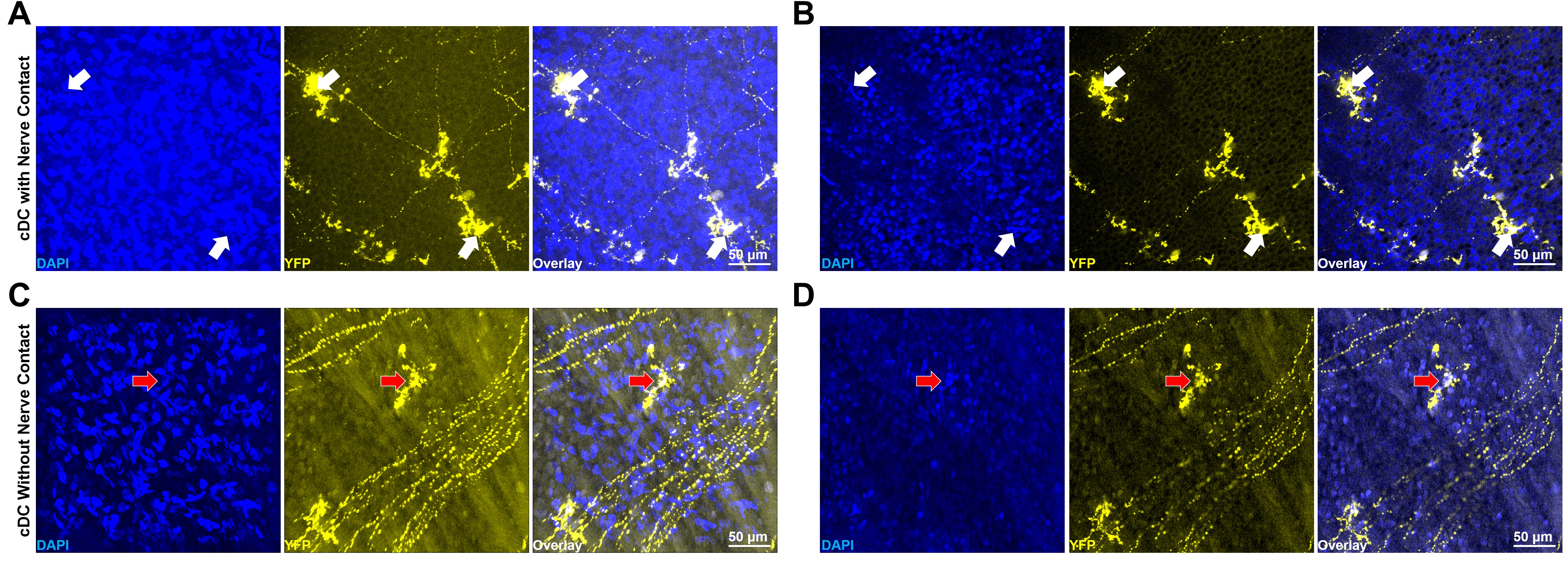

Supplement: FIGURE S4 — Morphologic differentiation of conventional dendritic cells and nerves in the cornea of transgenic CD11cYFP × Thy1YFP mice. Representative confocal micrograph of freshly excised corneal/limbal sample from a naïve CD11cYFP × Thy1YFP mouse mounted with DAPI. conventional dendritic cells (cDCs) exhibit a distinct morphology compared with nerves. (A) Representative maximum intensity projection of whole-mounted tissue, depicting cDCs in contact with nerves (white arrows). (B) Respective section on the level of nucleus shows presence of nucleus in the YFP+ cDCs (white arrows). (C) Representative maximum intensity projection of whole-mounted tissue, illustrating cDCs without contact with nerves (red arrow). (D) Respective section on the level of nucleus shows presence of nucleus in the YFP+ cDC (red arrow). The experiment was repeated 3 times. Scale bars: 50 μm. [file Image_4.JPEG]

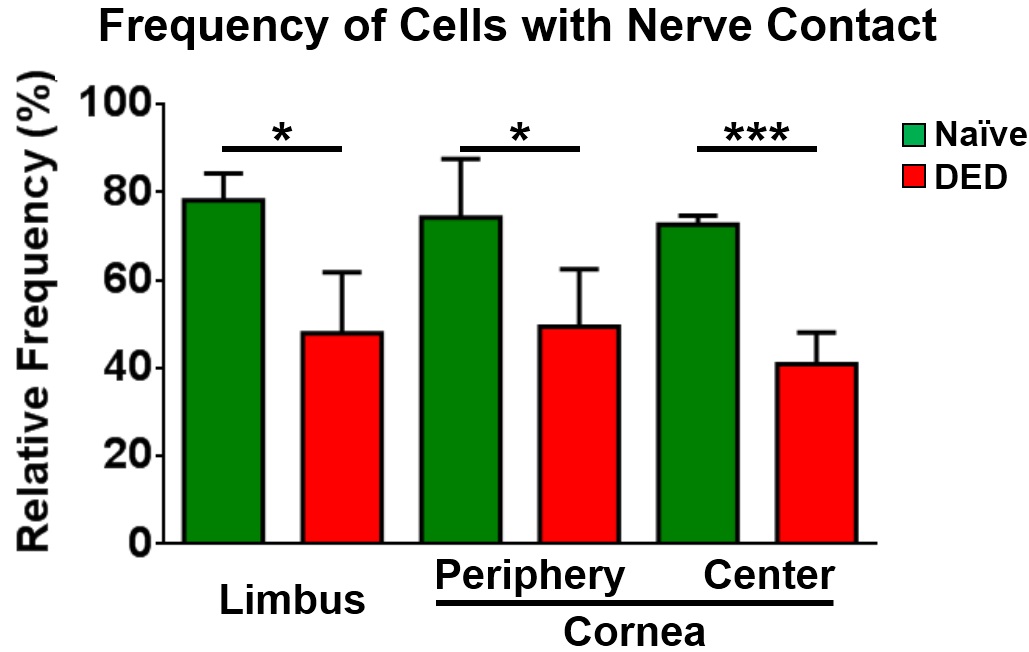

Supplement: FIGURE S5 — Frequency of conventional dendritic cells in contact with nerves in the limbus and cornea of transgenic CD11cYFP × Thy1YFP mice. Quantification of relative frequency of YFP+ conventional dendritic cells in contact with nerves in the limbus, peripheral, and central cornea in naïve mice and following exposure to desiccating stress (n = 3–5/group). Results are presented as mean ± SEM, t-test, ∗p < 0.05, ∗∗∗p < 0.001. [file Image_5.jpg]
